# A deep learning‐enabled toolkit for the 3D segmentation of ventricular cardiomyocytes

**DOI:** 10.1113/JP288557

**Published:** 2025-10-01

**Authors:** Joachim Greiner, Fabio Frangiamore, Frédéric Sonak, Josef Madl, Thomas Seidel, Peter Kohl, Eva A. Rog‐Zielinska

**Affiliations:** ^1^ Institute for Experimental Cardiovascular Medicine, University Heart Center Freiburg · Bad Krozingen, and Faculty of Medicine University of Freiburg Freiburg im Breisgau Germany; ^2^ CIBSS Centre for Integrative Biological Signalling Studies University of Freiburg Freiburg im Breisgau Germany; ^3^ Orobix Srl Bergamo Italy; ^4^ Institute of Cellular and Molecular Physiology Friedrich‐Alexander‐Universität Erlangen‐Nürnberg Erlangen Germany

**Keywords:** 3D reconstruction, cardiac tissue microstructure, cardiomyocyte, segmentation, wheat germ agglutinin

## Abstract

**Abstract:**

Segmentation of cardiomyocytes in microscopic 3D volumes is key to our understanding of cardiac (patho‐)physiology; however, it poses substantial experimental and analytical challenges. Therefore, researchers often resort to inferring 3D information from 2D segmentations, which can lead to biased and incorrect conclusions. Deep learning‐based methods are showing promise with respect to robustly segmenting objects in volumes acquired using various imaging modalities; yet, they have not been applied to high‐resolution 3D cardiomyocyte segmentations, and suitable open‐source tools and datasets are lacking. Here, we present a deep learning‐enabled toolkit for segmentation of individual cardiomyocytes in 3D confocal microscopy volumes. We include a dataset of 73 volumes with expert annotations, covering seven species, including mouse, human, and elephant, and containing samples generated under different experimental conditions, such as post‐myocardial infarction and *ex vivo* slice cultures. The toolkit additionally contains an image restoration workflow to address imaging‐related artefacts, such as spatially varying blur. Our automatic cardiomyocyte segmentation workflow achieved an adapted Rand error of 0.063 ± 0.034 (∼94% voxel‐pair agreement) on the test set. Our semi‐automatic workflow reached a throughput of 3 cells min^−1^ on a challenging, previously unseen dataset. The toolkit and data are open‐source and accessible through a dedicated graphical user interface. In summary, we provide an accessible toolkit enabling researchers to extract quantitative data on cardiomyocyte microstructure from 3D confocal image stacks of cardiac tissue. Given the size and diversity of our dataset, we expect our methods to perform well across species and experimental conditions, facilitating high‐quality 3D reconstructions of large numbers of individual cardiomyocytes.

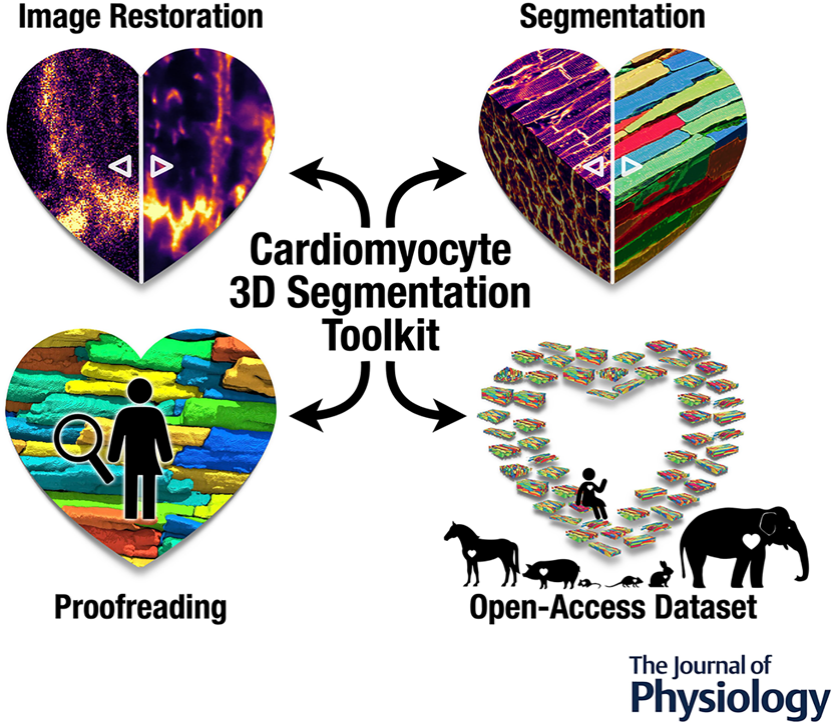

**Key points:**

- 3D cardiomyocyte microstructure is a key determinant of cardiac function in health and disease. However, reliable extraction and quantification of 3D cardiomyocyte cytoarchitecture pose significant experimental and computational challenges.
- We present an effective experimental protocol and a deep learning‐enabled toolkit for sample preparation and 3D analysis of cardiomyocyte morphology in ventricular myocardium.
- Our method is validated across seven species (mouse to human) and in samples prepared in diverse experimental conditions from a range of models, including myocardial infarction and *ex vivo* tissue culture, highlighting the robustness and versatility of our workflow.
- Our open‐source dataset and toolkit enable large‐scale analyses and extraction of realistic 3D geometries of ventricular microstructure. These can be used to explore a host of research questions and provide a new resource for modelling cardiac function at the cellular level.

## Graphical abstract

**Abstract figure legend** 3D cardiomyocyte segmentation enables comprehensive analyses of myocardial microstructure in health and disease; however, it is technically demanding. We present an open‐source toolkit for this task, which reduces challenges associated with sample preparation, image restoration, segmentation and proofreading. Alongside, we release a unique dataset containing 73 confocal microscopy image volumes with 3D cardiomyocyte annotations from seven species (including humans), acquired using different microscopes across several institutions. The datasets sample ventricular myocardium from control, post‐myocardial infarction, and *ex vivo* slice culture conditions, capturing a broad biological scope. On test data, our automatic workflow achieves an adapted Rand error of 0.063 ± 0.034, which can be quickly corrected using our proofreading tool, enabling high‐quality 3D reconstructions of cardiomyocyte microstructure.

## Introduction

In a healthy heart, most of its volume is occupied by muscle cells (cardiomyocytes). Pathological remodelling of cardiomyocytes is observed in chronic heart diseases, including heart failure, myocardial infarction and atrial fibrillation (Heijman et al., 2014; Schaper et al., 2002; Sutton & Sharpe, 2000). The process of delineating cardiomyocytes in image data, known as cardiomyocyte segmentation, is an important task to understand the structural basis of cardiac (patho‐)physiology. For those who are unfamiliar with image segmentation terminology, a plain‐language glossary of key terms is provided in the Appendix (Table A1). 3D cardiomyocyte segmentation is challenging because of their complex geometries and tight packing. This challenge is further amplified when analysing extended tissue volumes, where photon scattering and declining signal‐to‐noise ratios hinder fully automated segmentation.

**Figure 1:**
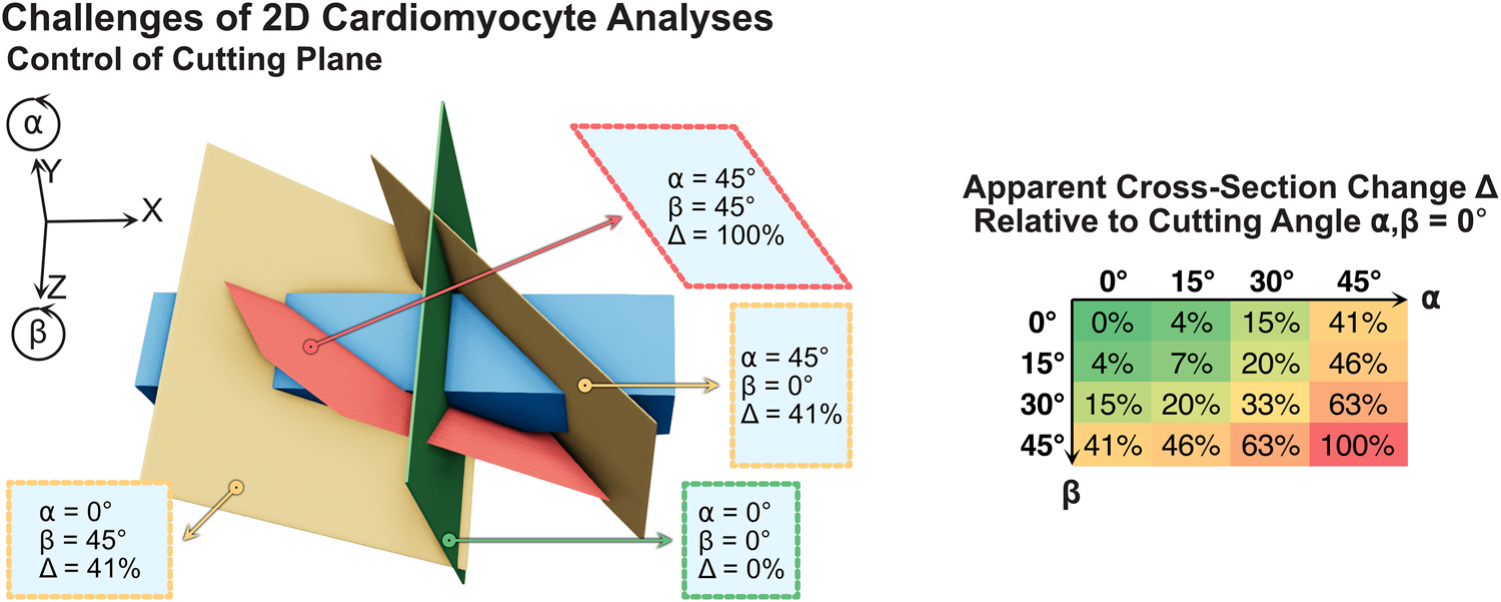
Challenges of 2D cardiomyocyte analyses, related to limited control over the cutting plane 2D analyses of cardiomyocytes often assume a perfectly orthogonal cutting plane, relative to one of the principal axes of the cell. Here, with the help of a simple 3D block, we demonstrate the effect of non‐orthogonal cuts on the determined apparent cross‐sectional area, using a numerical simulation. Of note, orientation of cardiomyocytes in tissue can change quickly over a distance as short as 100 µm, and so it is almost impossible to cut all cardiomyocytes within a given field of view in a uniform manner.

Consequently, researchers often use 2D imaging and analyses to infer 3D information − relying, for example, on stereological analysis (Brüel & Nyengaard, 2005), or automatic 2D segmentation tools such as JavaCyte, MARTA and CmyoSize applied to thin (∼5 µm) tissue sections (da Silva et al., 2022; Oliver‐Gelabert et al., 2020; Winters et al., 2020). However, these approaches have severe limitations because cardiomyocyte structure and organisation are inherently 3D. For example, assessment of cardiomyocyte cross‐sectional area (e.g. as a measure of hypertrophy) or sarcomere length (to characterise the mechanical state at the time of fixation of cardiomyocytes) relies on 2D sections that run in a defined plane relative to the cell geometry (perpendicular to the long axis of a cell for cross‐sectional area; parallel to contractile filaments for sarcomere length). Cutting tissue at a pre‐determined angle relative to its cellular make‐up is challenging for native tissue, and perhaps even impossible, as the cardiomyocyte orientation changes across the ventricular wall. Thus, reliance on 2D analysis may lead to overestimation of distances or areas (cosine error) (Fig. 1), potentially leading to incomplete or erroneous conclusions (Bub et al., 2010). By contrast, 3D reconstructions of cardiomyocytes are a powerful method for characterising healthy and diseased myocardium, and they are essential for developing robust conceptual and computational models.

Early work on 3D cardiomyocyte segmentation used manual deformation of surface meshes, followed by iterative thresholding (Lasher et al., 2009). Interactive deformation is time‐consuming, with a total workload of 1–2 weeks per image volume (Seidel et al., 2013). Subsequent studies replaced the mesh deformation with watershed‐based partitioning and manual agglomeration, reducing the required effort to 5–10 h for a similarly sized image volume (Greiner et al., 2018; Seidel et al., 2013). Parallel efforts achieved an estimated reconstruction throughput of 300–400 cardiomyocytes per workday in 40 µm thick paraffin sections, using a commercially‐available analysis software (Bensley et al., 2016). However, as a result of the processing steps for paraffin sections, capillary collapse and tissue shrinkage (e.g. during sample dehydration) may have accentuated cardiomyocyte boundaries. Additionally, the focus of prior work was on fully contained cardiomyocytes, rather than on reconstructing all cardiomyocytes in a given tissue volume, which may have eased the segmentation effort compared to whole‐volume reconstructions of non‐dehydrated, compression‐free mounted tissue (Seidel et al., 2016).

Overall, however, the development of methods for 3D cardiomyocyte reconstructions is lagging behind recent advances in 3D data acquisition. This is also reflected in a lack of established, publicly available methods and datasets for this task. Applying methods from other fields may involve substantial fine‐tuning, computational skills or resources that might not be available to interested researchers. To address these challenges, we developed and validated a comprehensive, deep learning‐enabled toolkit for 3D instance reconstruction of cardiomyocytes in confocal microscopy volumes, and introduce an interactive segmentation tool with a graphical user interface called ‘SegmentPuzzler’. This tool facilitates fast annotation and proofreading of segmentations based on clustered volumetric pixels known as ‘supervoxels’. Additionally, we provide a publicly accessible dataset of 3D cardiomyocyte segmentations. The diversity and size of this dataset make it valuable for future development of robust deep learning‐based models. It has been obtained using a time‐ and cost‐effective sample preparation protocol, which is suitable for various species and conditions. Given the large size of cardiomyocytes (up to 400 µm^2^ in cross‐sectional area), larger image volumes are beneficial because they probably contain several cell‐layers. As our sample preparation protocol relies on passive matching of refractive indices, some of the acquired stacks, especially those exceeding a depth of 100 µm, are affected by photon scattering‐induced, spatially varying blur and noise. To address this, we developed an image restoration method, which uses a novel approach to generate paired volumes with different signal intensities, blur and background as training data. All code and data are publicly available, and we provide a graphical user interface (GUI) for our tools.

## Methods

### Ethical approval

All datasets were sourced with ethical approval from the respective institutions. Mouse, rat, rabbit and pig experiments were carried out according to the guidelines in Directive 2010/63/EU of the European Parliament on the protection of animals used for scientific purposes and were approved by local authorities in Baden–Württemberg, Germany (X‐16/10R, TVA G19–160, X19/01R and X‐20/05R). Animals were maintained under a 12:12 h light/dark photocycle at room temperature with food and water *ad libitum*. All animal procedures and housing were handled according to the guidelines of the European Community's Council Directive of 22 September 2010 (2010/63/EU) and were approved by the regional council (Regierungspräsidium Freiburg). Cardiac tissue from horses and elephant was sourced from the diagnostic pool of the Institute of Veterinary Pathology at Justus‐Liebig‐University Giessen (animals were either killed in accordance with animal welfare regulations because of their previous illnesses, or had died of natural causes).

Human cardiac tissue datasets had been acquired by the Intermountain Donor Services at the University of Utah, USA, with ethical approval and informed consent, as previously published (Seidel et al., 2017).

### Dataset I: left ventricular rabbit myocardium after myocardial infarction and *ex vivo* tissue culture

The dataset comprises image volumes of wheat germ agglutinin (WGA)‐labelled left‐ventricular tissue slices of New Zealand White rabbits (2.5–3.0 kg body weight) with and without myocardial infarction that were acquired using a SP8 microscope (Leica, Wetzlar, Germany) with a 40× oil objective (Table 1) (myocardial infarction; Greiner et al., 2018, 2022). Of note, WGA effectively stains the glycocalyx and can therefore be used to visualise cardiomyocyte outlines in tissue. Additionally, we included WGA‐labelled image volumes of tissue slices from left ventricular myocardium of female New Zealand White rabbits (age 10–16 weeks, 2.0–3.5 kg body weight) after acute and 7 day *ex vivo* tissue culture (i.e. contracting cardiac tissue slices cultured inside an incubator at 37 °C, subjected to mechanical preload and electrical stimulation (Table 1) (ex vivo tissue culture; Baron et al., 2024). Tissue culture image volumes were obtained using an LSM 780 confocal microscope (Zeiss, Oberkochen, Germany) with a 63× oil objective.

**Table 1:** Datasets of annotated WGA‐labelled left‐ventricular myocardium.

id	Species	Institution	Hardware	Number of stacks	Average volume (µm^3^)	Voxel size (nm^3^)
Myocardial infarction	Rabbit	University of Utah, USA	Leica TCS SP8, 40× oil	20	201 × 196 × 48	200 × 200 × 200
*Ex vivo* tissue culture	Rabbit	University of Erlangen, Germany	Zeiss LSM 780, 63× oil	27	150 × 150 × 20	100 × 100 × 200
Healthy multispecies	Mouse, rat, rabbit, pig, horse, elephant	University of Freiburg, Germany	Leica TCS SP8 X, 63× glycerol	24	322 × 237 × 46	(166–227)^2^ × 180
Healthy human	Human	University of Utah, USA	Leica TCS SP8, 40× oil	2	205 × 205 × 54	200 × 200 × 200

*Note*: Characteristics of curated datasets. To increase robustness, datasets were acquired using various protocols, microscopes, objectives and imaging parameters. All datasets use the lectin wheat germ agglutinin (WGA) to label the glycocalyx of left‐ventricular tissue, and therefore, indirectly, label cardiomyocyte outlines. The third dimension given for volume and voxel measures the *z*‐axis dimension (depth, parallel to light path).

### Dataset II: multispecies myocardium

We compiled a new dataset, focusing on multispecies reconstructions of adult left ventricular myocardium (Table 1) (healthy multispecies), including wild‐type C57BL/6 mice (8–12 weeks, *N* = 1 animal, *n* = 3 stacks), wild‐type Sprague–Dawley rats (male, 8–9 months, *N* = 3, *n* = 5), New Zealand White rabbits (∼60 days, ∼2.0 kg; *N* = 2, *n* = 2), adult Landrace pigs (male, ∼110 kg; *N* = 2; *n* = 7), horses (12 years; one male pony, 364 kg, one female Warmblood, 688 kg; *N* = 2, *n* = 6) and an Asian elephant (∼48 years, female, >1000 kg; *N* = 1, *n* = 1). Rat tissue samples were a gift from the Department of Stereotactic and Functional Neurosurgery, University of Freiburg. Horse and elephant tissue samples were obtained from the tissue bank of the Institute of Veterinary Pathology, Justus–Liebig–University Giessen.

Mice were killed by cervical dislocation. Rats were terminally anaesthetised by an i.p. injection of 10% ketamine (Bela‐Pharm GmbH & Co. KG, Vechta, Germany; 1 mL kg^−1^ body weight) and 2% xylazine (Rompun, Bayer‐Leverkusen, Germany; 0.4 mL kg^−1^ body weight). Pigs were anaesthetised with midazolam (0.5 mg kg^−1^ body weight) and ketamine (20 mg kg body weight) via i.m. injection, and killed with a lethal dose of propofol (10–20 mg kg^−1^ body weight) i.v. Rabbits were anaesthetised via i.m. injection of esketamine hydrochloride (Ketanest S 25 mg mL^−1^; Pfizer Pharma PFE GmbH, Berlin, Germany; 12.5 mg kg^−1^ body weight) and 2% xylazine hydrochloride (Rompun 2%; Bayer Vital GmbH, Leverkusen, Germany; 0.2 mL kg^−1^ body weight). Thiopental (Thiopental Inresa 0.5 g; Inresa Arzneimittel GmbH, Freiburg, Germany; 12.5 mg mL^−1^) was then injected via the ear vein until apnoea. Mouse, rat and rabbit hearts were fixed via coronary perfusion, using 4% paraformaldehyde. Pig tissue fragments of ∼1 cm^3 ^ size were fixed via immersion in 4% paraformaldehyde. Horse samples of approximately ∼1 cm^3 ^ size were fixed via immersion in 2% paraformaldehyde. Elephant tissue samples had been immersion‐fixed in 4% formalin, dehydrated, embedded in paraffin, and cut at a thickness of 80 µm.

Sample processing was adapted from our previous work (Greiner et al., 2018). In brief, except for elephant tissue, tissue samples were cut into cubes with a cross‐sectional area of ∼0.8 × 0.8 cm. The cubes were embedded in 4% low melting agarose (#6351.2; Carl Roth, Karlsruhe, Germany) and glued with the epicardium facing the sample holder of a vibratome (7000 smz; Campden Instruments Ltd, Loughborough, UK) using cyanoacrylate glue. Sections (100–300 µm thick) were cut using blade displacements of 1.5 mm amplitude at a frequency of 60 Hz, with an advance speed of 0.05 mm s^−1^. Slices were carefully freed from remaining agarose using forceps or brushes and then transferred into a 12‐well plate (#20261500; Greiner Bio‐One, Kremsmünster, Austria) containing phosphate‐buffered saline (PBS, pH 7.4; containing in mm: 137 NaCl, 2.7 KCl, 8.1 Na_2_HPO_4_ and 1.5 KH_2_PO_4_). The elephant paraffin sections were de‐waxed and serially rehydrated to PBS. Then, all tissue slices were incubated with 0.5% Triton X‐100 in PBS for 30 min at room temperature on an orbital shaker to improve subsequent stain penetration. Next, slices were washed three times using PBS supplemented with 0.01% Tween‐20. Slices were then incubated at least overnight and up to 48 h at 37°C with WGA‐CF488A (10–40 µg mL^−1^; #29022; Biotium, Hayward, CA, USA) on an orbital shaker. After three washes, the samples were mounted on cover glasses with FluoroMount G (#20242950; Thermo Fisher Scientific, Waltham, MA, USA) using a compression‐free mounting approach for improved microstructure preservation (Seidel et al., 2016). Samples were cured in a humidity‐controlled, air‐tight chamber for 1–3 days before sealing them with nail polish. Departing from our previously reported sample preparation protocol, we used an oversaturated potassium carbonate solution, which equilibrates to a relative humidity of 43% and remains stable over a wide range of temperatures, as means of humidity control. A TCS SP8 X laser scanning confocal microscope (Leica) with a 63× glycerol objective was used for imaging. To acquire 3D volumes, 1024 × 1024 pixel 2D images with pixel sizes of (90–180 nm)^2^ were recorded as a *z*‐stack series with 90–180 nm *z*‐spacing. We linearly increased laser power with acquisition depth during imaging to reduce scattering‐induced signal intensity attenuation. To increase the imaged tissue volume, up to three *z*‐stack tiles with 10% tile overlap were recorded. Tile scan stitching was performed using LAS X, version 3.5.7.23225 (Leica). Image volumes with a z‐spacing smaller than 115 nm were downsampled using a 2 × 2 × 2 kernel, resulting in *z*‐spacings of 180 nm.

### Dataset III: left ventricular human myocardium

We included two image volumes of ventricular myocardium from two female donors, aged 42 and 48 years, whose hearts were rejected for transplantation. The donors were individuals who had passed away from causes unrelated to cardiac diseases (Table 1) (healthy human; Seidel et al., 2017). These images were purposely excluded from neural network training, to assess our method's generalisability to unseen sample types.

### Restoration of images with spatially varying blur

We used the framework of CARE and Richardson‐Lucy networks and incorporated a new method to generate paired degraded and target image volumes (Chobola et al., 2023; Li et al., 2022; Weigert et al., 2018). To mimic the degradation of image quality at increased stack depths, we imaged the same volume with differently sized pinholes (0.6, 1, 2, 3, 4, 5 and 6 Airy Units; AU) and laser power intensities (differing by up to 200× between degraded and target images). We obtained image stacks of 1024 × 1024 × 32 voxels with an isotropic size of 180 × 180 × 180 nm on a SP8 X confocal microscope (Leica) with a 63× glycerol objective, as described for Dataset II. We curated 12 image volumes for the training and three as the testing set. During training, the network was tasked with predicting the 0.6 AU images based on given images with larger pinhole sizes, or with equal pinhole sizes and lower laser powers. We compared data‐driven network architectures to those that explicitly incorporate the image formation process in their architecture. For the data‐driven model, we adapted the official CARE code example for 3D denoising, which employs a small residual 3D U‐Net architecture of depth 2, resulting in 996,769 learnable parameters. We extracted 128 × 128 × 32 patches and trained the network for 100 epochs, minimising the mean absolute error as the loss function. For the image formation‐informed model, we adapted the LUCYD model (Chobola et al., 2023), totalling 24,964 learnable parameters. We added the same percentile‐based image normalisation strategy as in CARE to both degraded and clean images and extracted 64 × 64 × 32 patches. We trained the network for 200 epochs, minimising the average mean squared error (MSE) and the structural similarity index (SSIM) as the loss function.

For the evaluation, we normalised all images, as the dynamic range varies considerably between input and target images and within each image set. Following the strategy of CARE, we aligned predictions p and ground truth gt by normalising the images using a percentile‐based strategy as indicated before, obtaining $p_{\mathrm{norm}}$ and $gt_{\mathrm{norm}}$. We then optimised the multiplicative factor a and the offset b so that the MSE between scaled prediction $p_{\mathrm{scaled}}$ and $gt_{\mathrm{norm}}$ is minimised (Weigert et al., 2018):

(1) $$p_{\mathrm{scaled}}=a\cdot p_{\mathrm{norm}}+b$$

(2) $$a,b=\arg\min MSE\left(p_{\mathrm{scaled}},gt_{\mathrm{norm}}\right)$$

Then, we calculated the SSIM and the normalised mean square error (NMSE) on the test set.

To mitigate the effects of signal attenuation, we implemented a two‐step normalisation process. First, we estimated fluorescent foreground and background using a GPU‐accelerated 2D top‐hat filter with a radius of 25 voxels, applied to each *xy* slice, using the clEsperanto library (Haase et al., 2020). We calculated the 1% and 99% percentiles in the background and foreground images to obtain an estimate for the respective distribution. These resulting estimates were then smoothed along the *z*‐axis using a Savitzky–Golay filter with a window length of 101 (unitless index). Next, we determined image planes that were mostly void of fluorescence signal. To do so, we calculated a threshold value of 0.1 times the 90th percentile of the background‐subtracted input image for each image plane. The 1D threshold profile was then further filtered with a maximum filter of length 150 (unitless index) across the *z*‐axis to ensure that a valid threshold was derived, even if the complete image plane contained only background noise. Foreground and background values for image planes determined to be background noise were replaced using a value derived from nearest neighbour extrapolation. Finally, the original, uncorrected image was then normalised for each image plane, so that the background and foreground distribution cluster around the 5% and 30% values of the datatype's dynamic range, respectively.

### Automated cardiomyocyte segmentation

We compared three supervoxel‐based automatic segmentation pipelines: PlantSeg (Wolny et al., 2020), as well as two pipelines introduced by us, developed independently from each other, DTWS‐MC (distance‐transform watershed combined with the MultiCut algorithm) and DTWS‐MO (distance‐transform watershed combined with morphological operations).

PlantSeg is an automatic segmentation method originally developed for segmenting plant tissue at cellular resolution. We adapted the PlantSeg training recipe for boundary prediction in confocal volumes of *Arabidopsis* ovules. The 3D U‐Net was trained with the average of a binary cross‐entropy loss and a soft dice loss. PlantSeg applies a distance‐transform watershed to the thresholded predicted boundaries to generate supervoxels, and it offers several agglomeration strategies. We chose the default parameters for PlantSeg and added minor optimisations. First, we used a 3D watershed instead of a 2D watershed for supervoxel generation. Second, we optimised the MultiCut balancing parameter β to improve split/merge and segmentation metrics. To do so, we tested different values of β on a validation set (20% of the training data) and selected 0.8 because this value minimised the adapted Rand error (a common metric to assess instance segmentations based on pairwise comparisons of voxels, ranging from 0, indicating a perfect match, to 1, indicating complete disagreement). PlantSeg output provides a partitioned image containing both cardiomyocyte and background segments, whereas our cardiomyocyte annotation only included cardiomyocyte segments. To benchmark PlantSeg against our other methods, we added a foreground classification step. Segments were classified using a majority vote from a 3D U‐Net that predicted a semantic cardiomyocyte mask. The network was trained using the 3D configuration of the nnU‐Net framework (Isensee et al., 2021).

DTWS‐MC was developed based on our earlier watershed‐based segmentation workflow (Seidel et al., 2013). We added neural networks for boundary predictions and a MultiCut algorithm for supervoxel agglomeration, inspired by Beier et al. (2017). The annotation target of the network was cardiomyocyte boundaries (2 × dilated), with a two‐voxel layer ‘ignore layer’ placed around the boundary. The loss was masked within this layer and thus ignored during the training phase. This adjustment was introduced after we observed that most of the loss originated from the boundary interface, which is probably less critical than the boundary's overall presence. We used the 3D U‐Net configured from the nnU‐Net framework (Isensee et al., 2021), which also minimises a combination of the binary cross‐entropy loss and soft dice loss. For supervoxel generation, we used a parallelised block‐based distance‐transform watershed from the Python package elf (Pape et al., 2017). Next, we applied MultiCut agglomeration to generate instance proposals. As with the PlantSeg pipeline, we optimised the MultiCut balancing parameter, β, using the validation set and selected a value of 0.075. Similarly, cardiomyocyte segments were classified using the same 3D U‐Net that predicted a semantic cardiomyocyte mask as in our PlantSeg workflow.

DTWS‐MO used a 3D Attention U‐Net (Oktay et al., 2018) with five layers, trained using the mean of focal loss (Lin et al., 2020) and clDice loss (Shit et al., 2021) to predict a semantic mask of cardiomyocytes. The 3D Attention U‐Net used a patch size of 304 × 304 × 16 voxels. A prediction strategy with overlapping tiles (stride: 0.5) was used during inference. The predicted mask was binarised with a threshold value of 0.85. From the binarised mask, a Euclidean distance transform was computed, and values with a distance less than three voxels were set to the background. A morphological 3D opening was then applied using spherical structural elements with radii of six and eight voxels for cells smaller and larger than 10^6^ and 10^7^ voxels, respectively. Additionally, all small cells (< 5 * 10^3^ voxels) were excluded. A watershed transform was applied to this filtered mask to obtain the final segmentation.

We also investigated two generalist segmentation tools with strong performance on biological data: Cellpose (Stringer et al., 2021) and Omnipose (Cutler et al., 2022). We followed two 3D segmentation strategies proposed by the original Cellpose package, which we term Cellpose‐XY and Cellpose‐O. Cellpose‐XY consists of a single 2D Cellpose model trained on XY slices. Predictions were subsequently stitched across in 3D with a stitch_threshold of 0.1 (indicating a 10% overlap required for stitching). Cellpose‐O consists of two separately trained 2D Cellpose models: one exclusively trained on XY slices and another trained jointly on YZ and XZ slices. Predictions of orthogonal planes were combined and processed using the built‐in functions of Cellpose, allowing for 3D reconstruction of cardiomyocytes based on the combined information. For training, we randomly extracted 20 crops (512 × 512 pixels) from *XY* planes of each image stack. Similarly, 20 crops of size 512 × 100 pixels were extracted from each stack's *YZ* and *XZ* planes. Training was carried out from scratch without using pre‐trained weights. Input images were rescaled to an average cardiomyocyte diameter of 30 voxels for all models. Models were trained for 500 epochs using the recommended hyperparameter choices (learning rate of 0.2, stochastic gradient descent with momentum of 0.9, weight decay of 0.00001 and batch size of 8). We also trained a 3D Omnipose model. To allow the network to capture larger portions of cardiomyocytes in its receptive field, we downsampled the dataset by a factor of 3 in each dimension before training the model for 1000 epochs using reported hyperparameters (learning rate of 0.1, stochastic gradient descent with momentum of 0.9, weight decay of 0.00001 and batch size of 4). In our preliminary assessments, we evaluated StarDist as a potential general segmentation tool (Weigert & Schmidt, 2022). However, StarDist requires cell shapes to be sufficiently star‐convex, such that most points on the cell boundary should be reachable from a central point inside the cell by a straight‐line segment that remains entirely within the cell. We found that this requirement is often violated for cardiomyocytes. This fundamental limitation restricts segmentation accuracy for cardiomyocytes; therefore, we opted not to investigate the performance of StarDist in detail.

We benchmarked the segmentation models using the adapted Rand error and the variation‐of‐information split and merge terms (quantifying over‐ and under‐segmentation, respectively), all computed with scikit‐image (van der Walt et al., 2014).

### Semi‐automatic cardiomyocyte segmentation

**Figure 2:**
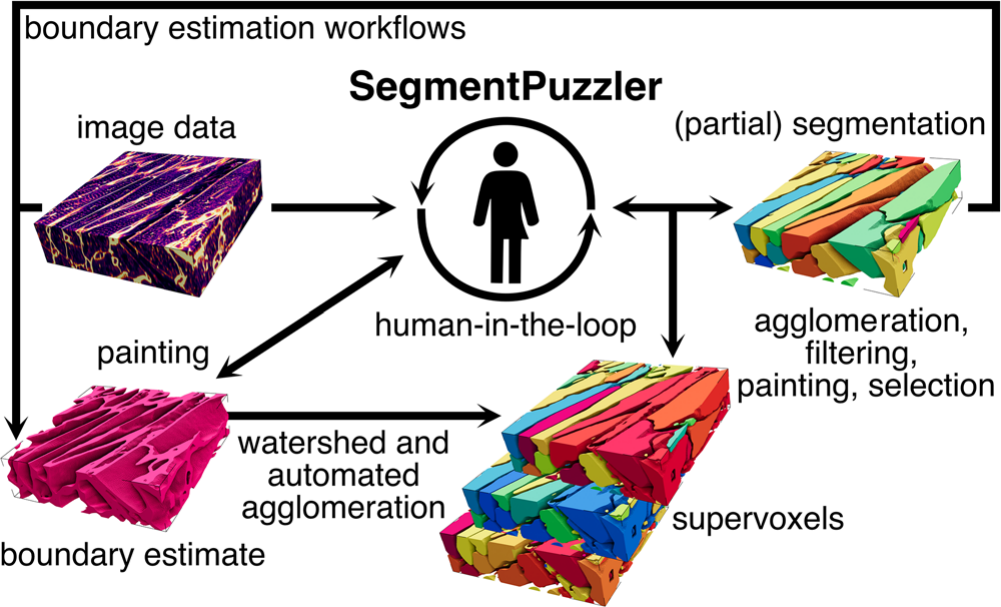
Overview of the ‘SegmentPuzzler’ workflow for semi‐automatic segmentation and annotation SegmentPuzzler enables users to interactively agglomerate, generate, modify and select supervoxels and segments into accurate segmentations.

To annotate newly acquired data and proofread existing ones, we developed the new open‐source program SegmentPuzzler (Fig. 2). SegmentPuzzler was written in C++ and its GUI is based on the open‐source Qt framework. Filters and image input/output are handled by the ITK library (McCormick et al., 2014). SegmentPuzzler displays image data in three orthogonal views in its primary interface. The user can load supervoxels and boundary image data (e.g. generated by neural networks, or random forests) (Berg et al., 2019). SegmentPuzzler can also generate supervoxels from boundary image data. Three major tools are provided: supervoxel agglomeration into segments, supervoxel (re‐)generation and segment selection. Agglomeration is the main task of SegmentPuzzler, enabling users to merge and split supervoxels rapidly. Using the GUI, the user can merge and split supervoxels by dragging the mouse with the left or right mouse button pressed, respectively. During this process, the user is informed by three orthogonal views, which can overlay arbitrary image volumes, such as multichannel fluorescence data and neural network predictions, to aid the segmentation process. Furthermore, the user can specify the colour and transparency of overlaid image volumes. We used SegmentPuzzler to proofread segmentations in all datasets.

### Cardiomyocyte segmentation application examples

We demonstrate possible downstream applications of our toolkit by calculating features assessing microstructural composition (myocyte volume fraction; WGA‐positive extra‐myocyte volume fraction), cellular morphology (cardiomyocyte length, width, height, cross‐sectional area, volume and count) and subcellular structure (distance of the cytoplasm to the nearest voxel of the transverse‐axial tubular system; TATS).

Myocyte volume fraction was defined as the fraction of voxels that were segmented as cardiomyocytes. For the determination of the WGA‐positive extra‐myocyte space and TATS segmentation, WGA was automatically thresholded using the Li method within the scikit‐image library (van der Walt et al., 2014). Notably, because we include TATS in our cardiomyocyte segmentation masks, the WGA‐positive extra‐myocyte space also excludes the TATS‐associated WGA signal. For TATS thresholding, the WGA signal was additionally filtered using a GPU‐accelerated 2D top‐hat filter (radius = 5 voxels), applied to each *xy* slice, using the clEsperanto library (Haase et al., 2020). Mean and median distances of the cytoplasm to the nearest TATS voxel were calculated using the SignedMaurerDistanceMap function in ITK (McCormick et al., 2014). For morphological features, an oriented bounding box was fitted to each cardiomyocyte. Cardiomyocyte length was defined as the largest dimension of the oriented bounding box. Accordingly, width and height were calculated using the average extent along the second and third largest dimensions. Cross‐sectional area was derived as the average cross‐sectional area perpendicular to the largest dimension. Cardiomyocyte volume was determined by multiplying the number of voxels in the cardiomyocyte segmentation by the physical voxel volume. Cell count included all cardiomyocytes with a volume larger than 3 µL, comprising an empirically chosen threshold that reduces ambiguities caused by cell fragments at the image volume borders. To assess whether the analysis process was correctly implemented, we generated a block of known length, width, and height, and checked the generated errors for the calculated length, width, height, cross‐sectional area and volume for different block orientations. Errors were negligible with values not exceeding 10^−10^ µm (probably arising from the floating‐point number representation).

### Statistical evaluation and visualisation

The algorithms were evaluated on a representative test set of 14 stacks: four from the dataset ‘myocardial infarction’, four from the ‘slice culture’ dataset and six from the ‘multispecies’ dataset, with stratification for the chosen conditions/species. Statistical testing was done with the Python package pingouin (Vallat, 2018). Algorithms were compared using paired *t* tests and (repeated measures) ANOVA, where applicable. Following a significant ANOVA result, pairwise comparisons were conducted using *post hoc* tests with Bonferroni–Holm corrections to control for multiple comparisons. Effect size was calculated using Cohen's *d*, where we classify effect sizes as small (0.2 ≤ *d* < 0.5), medium (0.5 ≤ *d* < 0.8) and large (*d* ≥ 0.8), respectively. We used BokehBioImageDataVis (https://github.com/JoeGreiner/BokehBioImageDataVis) to generate interactive web visualisations.

## Results

### 3D cardiomyocyte dataset curation

**Figure 3:**
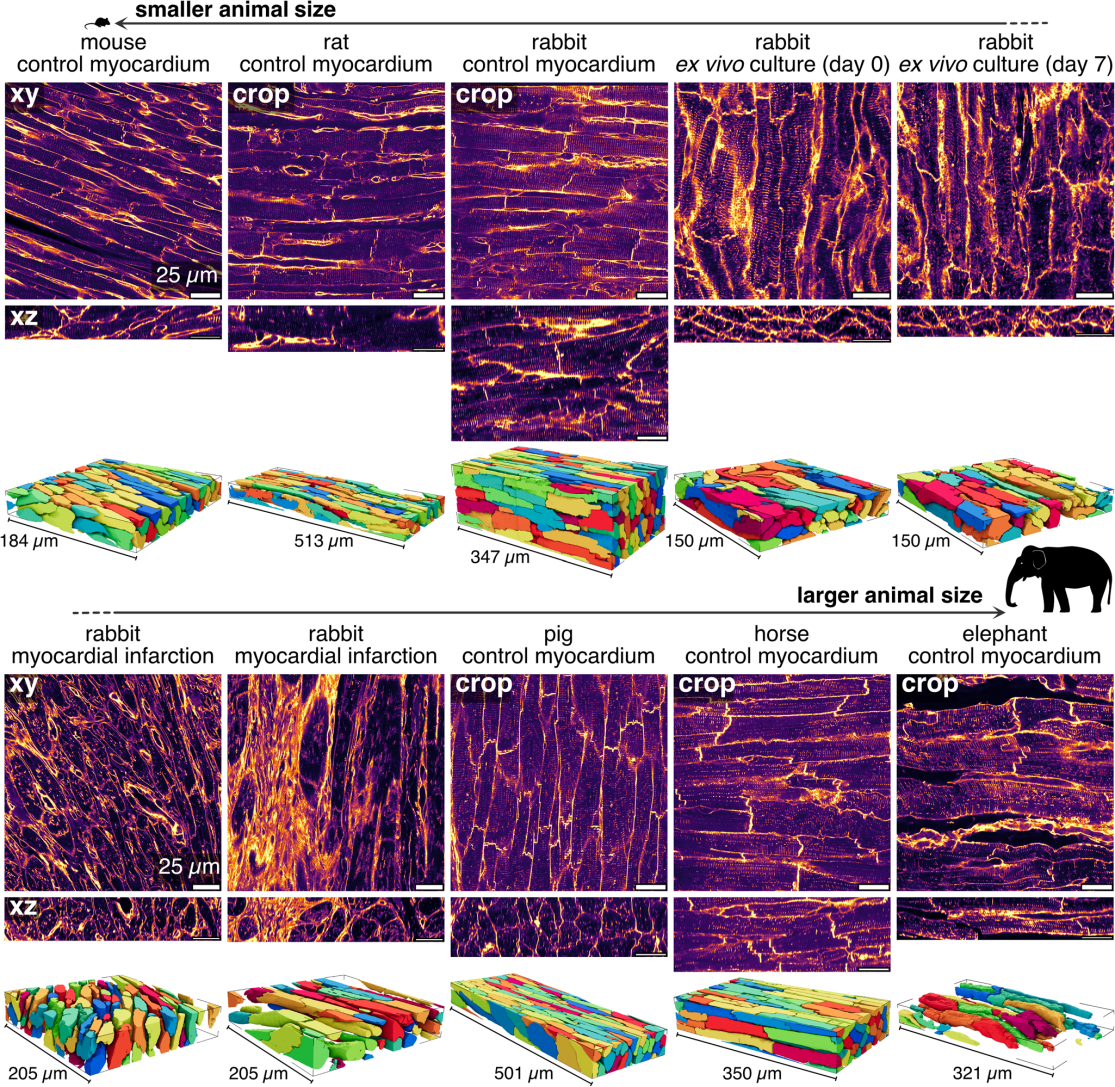
3D segmentation and *xy*/*xz* views of WGA‐labelled myocardium across diverse experimental conditions and species, including control myocardium, myocardium post‐myocardial infarction and myocardium after *ex vivo* tissue culture for 7 days For large volumes, cropped images are shown in XY. All image stacks with their corresponding annotation used in the present study are visualised in the Supplementary Web Visualisation S1. We recommend inspecting the web visualisations in addition to the static version above for exploration of image and annotation data. The visualisation can be viewed in a browser without the need for dedicated software.

We curated a large and diverse dataset of image volumes of left ventricular myocardium labelled with WGA, containing 3D cardiomyocyte annotations generated with our semi‐automatic segmentation workflow SegmentPuzzler (Fig. 3 and Table 1). Manual effort heavily depended on stack size and image quality, generally ranging from 0.5 to 8 h per stack.

### Image restoration

**Figure 4:**
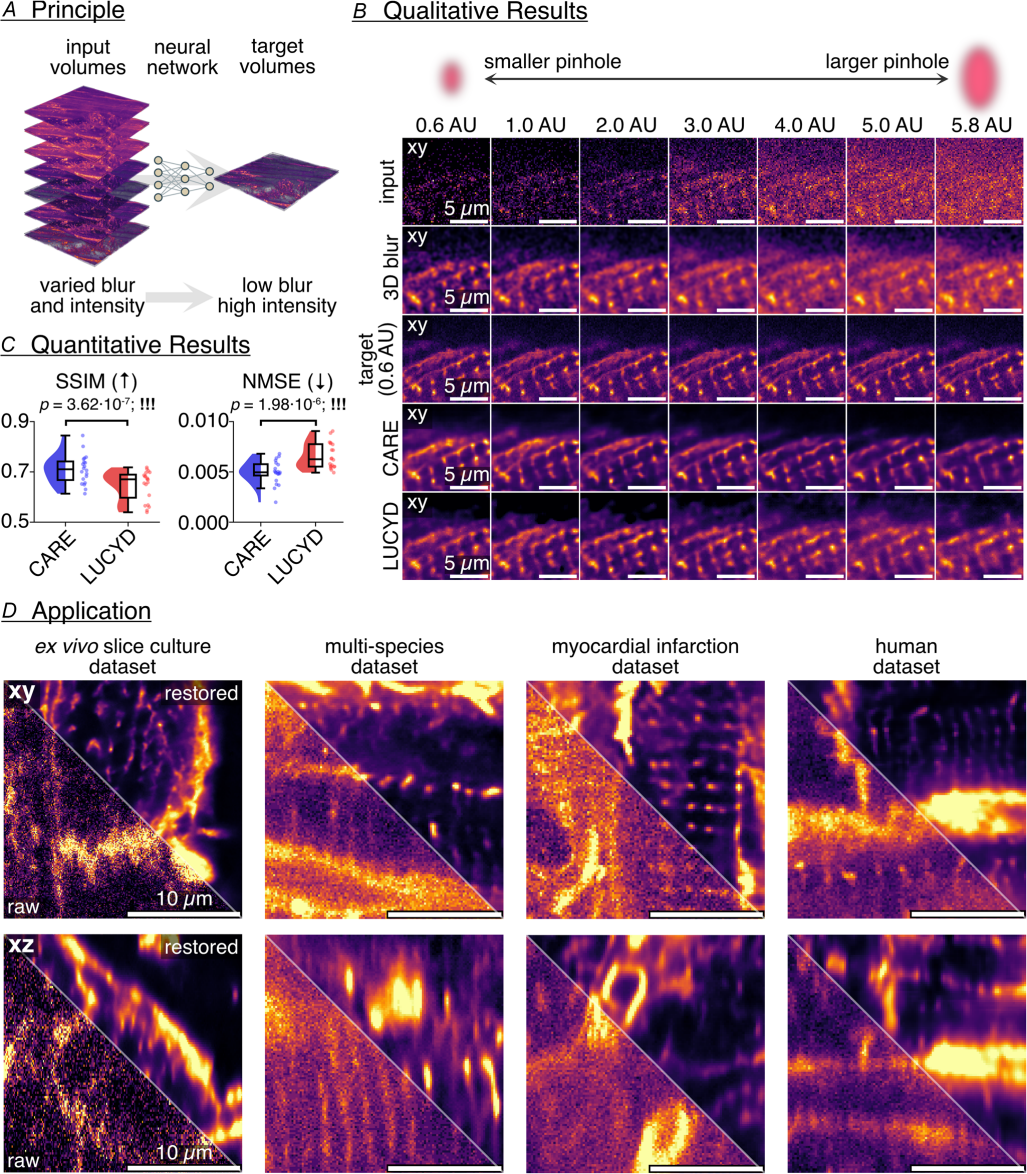
Training, evaluation and application of an image restoration method addressing spatially varying blur *A*, during the training process, the network is tasked with restoring image volumes with low signal intensity and a pinhole size of 0.6–6 Airy Units (AU) to image volumes of high signal intensity acquired with a pinhole size of 0.6 AU. *B*, qualitative results for a stack from the test set, acquired with very low laser power. Comparative reconstructions of a simple 3D Gaussian blur (sigma: 1), a data‐driven CARE model and a model based on the image formation process, LUCYD, are shown. Note that the target image is identical for all pinhole values. *C*, quantitative evaluation of the denoising models (*n* = 20 image volumes). Arrows indicate whether the metric should be maximised or minimised. Small, medium, and large effect sizes are indicated by ‘!’, ‘!!’ and ‘!!!’, respectively. *D*, application of the CARE model to datasets used in this study, shown in both *xy* and *xz* planes of volume crops. All raw and restored image stacks used in our study are visualised in the Supplementary Web Visualisation S2.

We evaluated the performance of our image restoration workflow for spatially varying blur using both qualitative and quantitative assessments (Fig. 4 and Table 2). Qualitatively, both the CARE and LUCYD models could reconstruct the gross microstructure from inputs with low signal‐to‐noise ratio and high blur (Fig. 4*B*). However, we observed superior performance in restoring finer structures and stronger robustness to high degrees of blur with the CARE model, compared to LUCYD (Fig. 4*B*, 4–5.8 AU). Quantitatively, the CARE model significantly outperformed the LUCYD model with large effect sizes in both SSIM (0.71 *vs*. 0.65, *P* = 3.6 × 10^−7^, *d* = 1.1; *n* = 20 image volumes; Fig. 4*C*, left) and NMSE (0.0050 *vs*. 0.0066, *P* = 2.0 × 10^−6^, *d* = 1.3, *n* = 20; Fig. 4*C*, right). When applying the CARE model to our study's datasets, we observed a strong denoising effect and notable improvements in image sharpness, particularly along the axial direction (Fig. 4*D*, see also Supplementary Web Visualisation S2).

**Table 2:** Test set segmentation metrics: image restoration.

Method	SSIM	NMSE
No processing	0.34	0.0163
LUCYD	0.64	0.0066
CARE	**0.71**	**0.0050**

*Note*: Best observed metric is indicated in bold. *n* = 20 image volumes.

Abbreviations: SSIM, structural similarity index; NMSE, normalised mean squared error.

### Automatic cardiomyocyte segmentation

**Figure 5:**
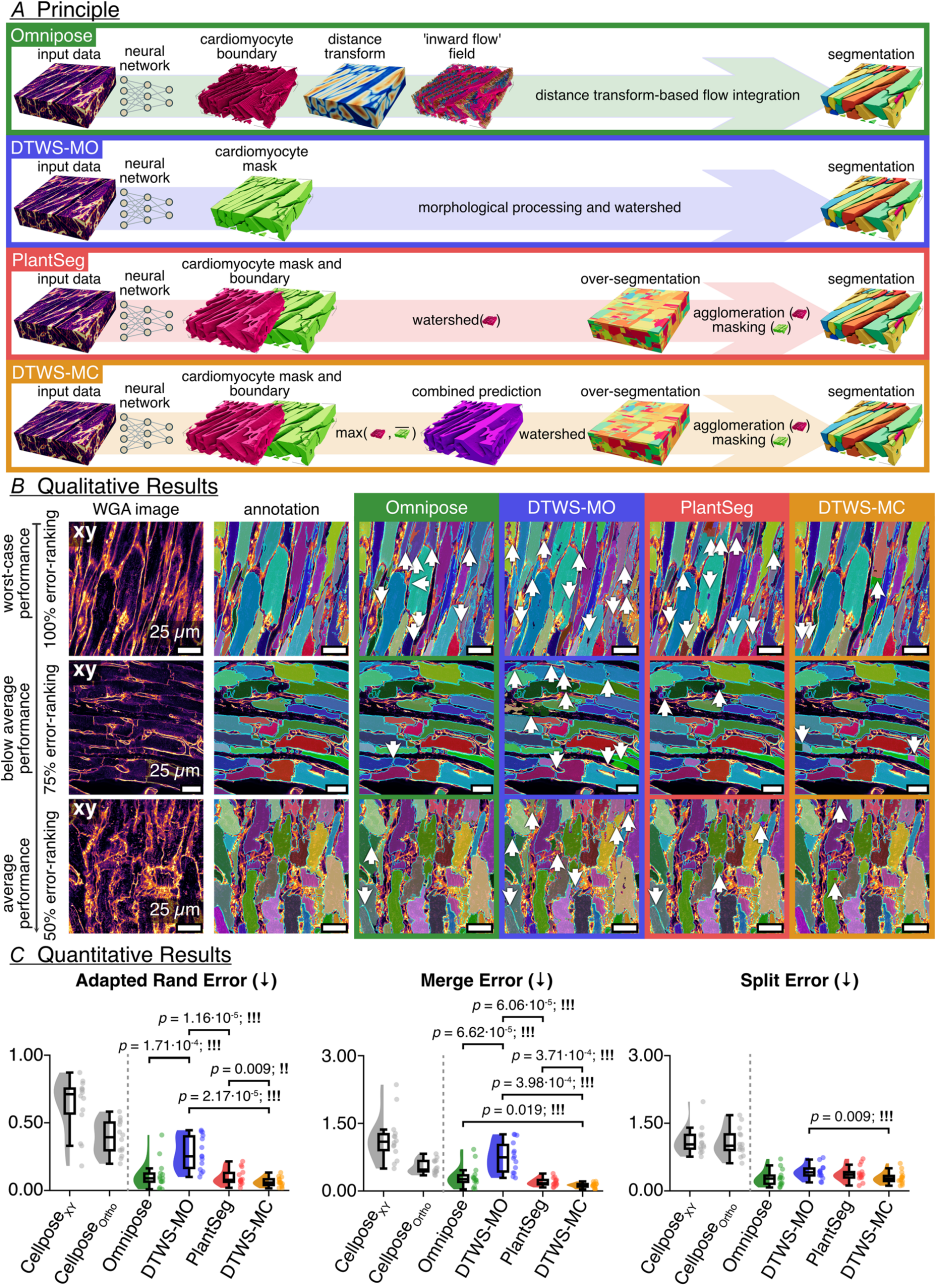
Comparison of automatic segmentation methods for cardiomyocytes *A*, overview of segmentation workflows of Omnipose, DTWS‐MO, PlantSeg and DTWS‐MC. *B*, qualitative evaluation investigating failure cases. White arrows highlight segmentation errors. *C*, quantitative evaluation of adapted Rand, merge and split errors (*n* = 14 image volumes). The results are presented with arrows indicating whether a metric should be maximised or minimised. Effect sizes are indicated by ‘!’, ‘!!’ and ‘!!!’ for small, medium and large effects, respectively. Cellpose models are shown for completeness but were excluded from the statistical analyses. All predictions on the test set of all three methods are visualised in the Supplementary Web Visualisation S3.

**Figure A1:**
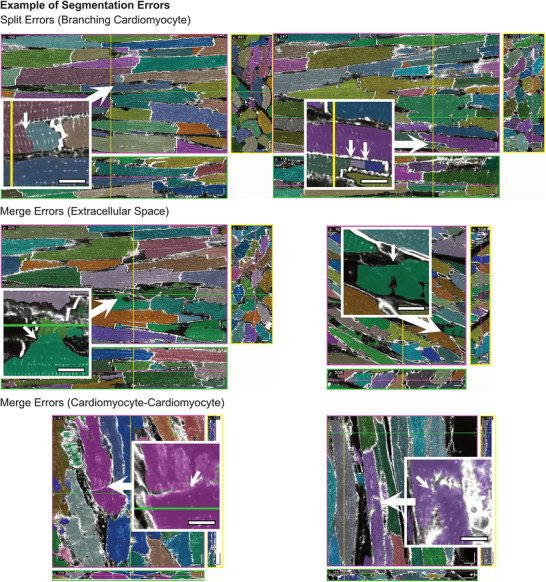
Examples of split and merge errors in the context of cardiomyocyte segmentation, taken from the Supplementary Web Visualisation S3 Split errors show examples where one cardiomyocyte has been wrongly assigned more than one segment. Merge errors (extracellular space) show cases where cardiomyocytes are incorrectly merged with extracellular space. Merge errors (cardiomyocyte‐cardiomyocyte) show examples where two different cardiomyocytes have been detected as a single cardiomyocyte. Arrows point towards the error location, with one error per image shown as a magnified inset. Scale bar = 10 µm.

Having established a large and diverse dataset and a strong image pre‐processing pipeline, we evaluated the performance of automatic cardiomyocyte segmentation methods using both qualitative and quantitative assessments (Fig. 5 and Table 3). The segmentation errors of Cellpose‐O and Cellpose‐XY were much higher than those of the other algorithms tested (Fig. 5*C*). Therefore, we show their results for completeness, without including them in further analyses. To ensure a fair qualitative assessment, we followed a rule‐based approach to generate representative images: First, we ranked every stack by the adapted Rand error for each method and then sorted by the average ranking across all methods (Fig. 5*B*). Then, we presented the average case (50% error ranking), a poor case (75% error ranking) and the worst case (highest error ranking). Stacks higher in this ranking, representing more challenging‐to‐segment stacks, originated predominantly from remodelled myocardium and/or suffered from suboptimal WGA labelling. In these stacks, we observed notable qualitative differences between methods: DTWS‐MO frequently produced segmentations with multiple over‐merged cardiomyocytes (merge errors) and frequent instances of cardiomyocytes falsely extending into extracellular space (also merge errors; Fig. 5*B*; see also Supplementary Web Visualisation S3). These errors were less often observed with DTWS‐MC, Omnipose and PlantSeg. Occasionally, DTWS‐MC and PlantSeg produced over‐split cardiomyocytes, especially in regions containing branching cardiomyocytes (split errors; see also Supplementary Web Visualisation S3). Additional examples of split and merge errors are illustrated in the Appendix (Fig. A1). Quantitatively, DTWS‐MC produced significantly better overall segmentations than both PlantSeg and DTWS‐MO, with medium to large effect sizes, as measured by the average adapted Rand error (0.06 *vs*. 0.10, *P* = 3.2 × 10^−3^, *d* = 0.7 for PlantSeg and 0.06 *vs*. 0.28, *P* = 8.7 × 10^−6^, *d = *2.3 for DTWS‐MO; *n* = 14 image volumes; Fig. 5*C*, left). We did not observe a statistically significant difference in adapted Rand error between DTWS‐MC and Omnipose (*P* = 0.125). We benchmarked the runtime of the three best‐performing algorithms (see Appendix, Table A2) on an image stack with 2778 × 1016 × 280 voxels. DTWS‐MC completed the task in 61 min (17 min without test‐time augmentation), PlantSeg completed the task in 15 min and Omnipose completed the task in 4 min.

**Table 3:** Test set segmentation metrics: automatic workflows.

Method	Adapted rand error	Merge error	Split error
Cellpose‐XY	0.63	1.16	1.11
Cellpose‐O	0.39	0.53	1.08
Omnipose‐3D	0.11	0.32	**0.29**
DTWS‐MO	0.28	0.76	0.42
PlantSeg	0.10	0.20	0.38
DTWS‐MC	**0.06**	**0.12**	**0.29**

*Note*: Best observed metric is indicated in bold. *n* = 14 image volumes.

To better understand segmentation errors, we examined probabilistic measures of split and merge errors (Fig. 5*C*, centre and right). On average, DTWS‐MO generated a higher number of merge errors than split errors, whereas PlantSeg and DTWS‐MC produced fewer merge errors compared to split errors. Omnipose generated approximately equal numbers of split and merge errors. DTWS‐MC produced significantly fewer merge errors with a large effect size (0.12 *vs*. 0.20, *P* = 1.2 × 10^−4^, *d* = 1.0 for PlantSeg; 0.12 *vs*. 0.76, *P* = 2.0 × 10^−5^, *d* = 2.5 for DTWS‐MO; and 0.12 *vs*. 0.32, *P* = 0.019, *d* = 1.1 for Omnipose; *n* = 14 image volumes). DTWS‐MC produced significantly fewer split errors than DTWS‐MO, with a large effect size (0.29 *vs*. 0.42, *P* = 0.019, *d* = 0.8). We could not detect a significant difference in split errors between DTWS‐MC and PlantSeg (0.29 *vs*. 0.38, *P* = 0.085), or between Omnipose and PlantSeg (0.29 *vs*. 0.38, *P* = 0.208).

### Proofreading effort and generalisation to unseen conditions

**Figure A2:**
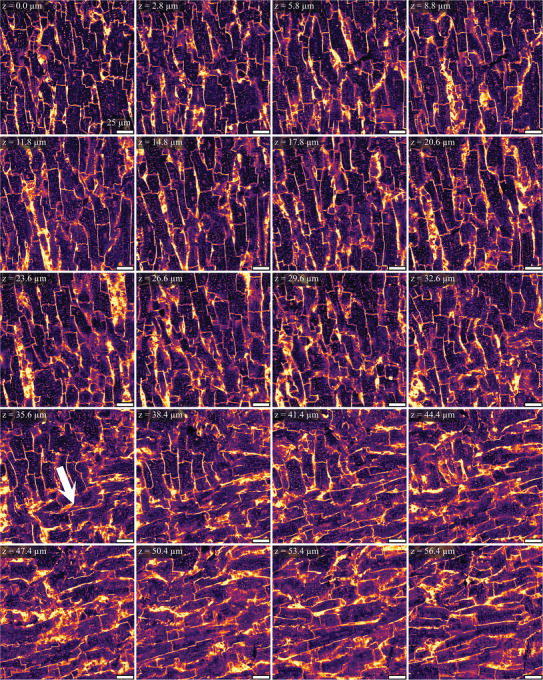
Example test data from healthy human ventricular myocardium (rejected donor hearts) The donors were individuals who had passed away from causes unrelated to cardiac diseases. A ∼90° cardiomyocyte orientation change starts around 35.6 µm (white arrow). Such cardiomyocyte orientation changes were not present in the training dataset. Scale bar = 25 µm.

Given the large variety of model organisms and experimental conditions under which cardiac research is conducted, we assessed to what extent our method, trained on datasets I and II, can be generalised to a new set of data, dataset III, and quantify the effort needed to proofread the segmentation using SegmentPuzzler. Dataset III contains images of a species not trained on (human), and sample preparation and imaging were performed by different researchers than for datasets I and II. To quantify proofreading effort and assess generalisation, we focused on the best‐performing DTWS‐MC method. Notably, cell morphology differed considerably from the other datasets, and one of the volumes showed an unusually abrupt 90° change of cardiomyocyte orientation in the centre of the volume (see Appendix, Fig. A2). The resulting segmentations were proofread using SegmentPuzzler, requiring 28 and 76 min for the two stacks containing 82 and 232 cardiomyocytes, respectively, resulting in a combined average throughput of 3 cells min^−1^. Comparing the initial segmentations with the corrected segmentations, the DTWS‐MC model resulted in an adapted Rand error of 0.06, a merge error of 0.09 and a split error of 0.07 (*n* = 2 image volumes).

### Downstream applications characterising cardiac (patho‐)physiology

Cardiomyocyte segmentation enables numerous downstream applications, including quantification of microstructural composition and fibrosis (Greiner et al., 2018), morphological analyses, subcellular protein/organelle/structure quantification and localisation (Lackey et al., 2011), as well as *in silico* experiments using realistic high‐resolution computational meshes (Greiner et al., 2018, 2022; Sankarankutty et al., 2021).

**Figure 6:**
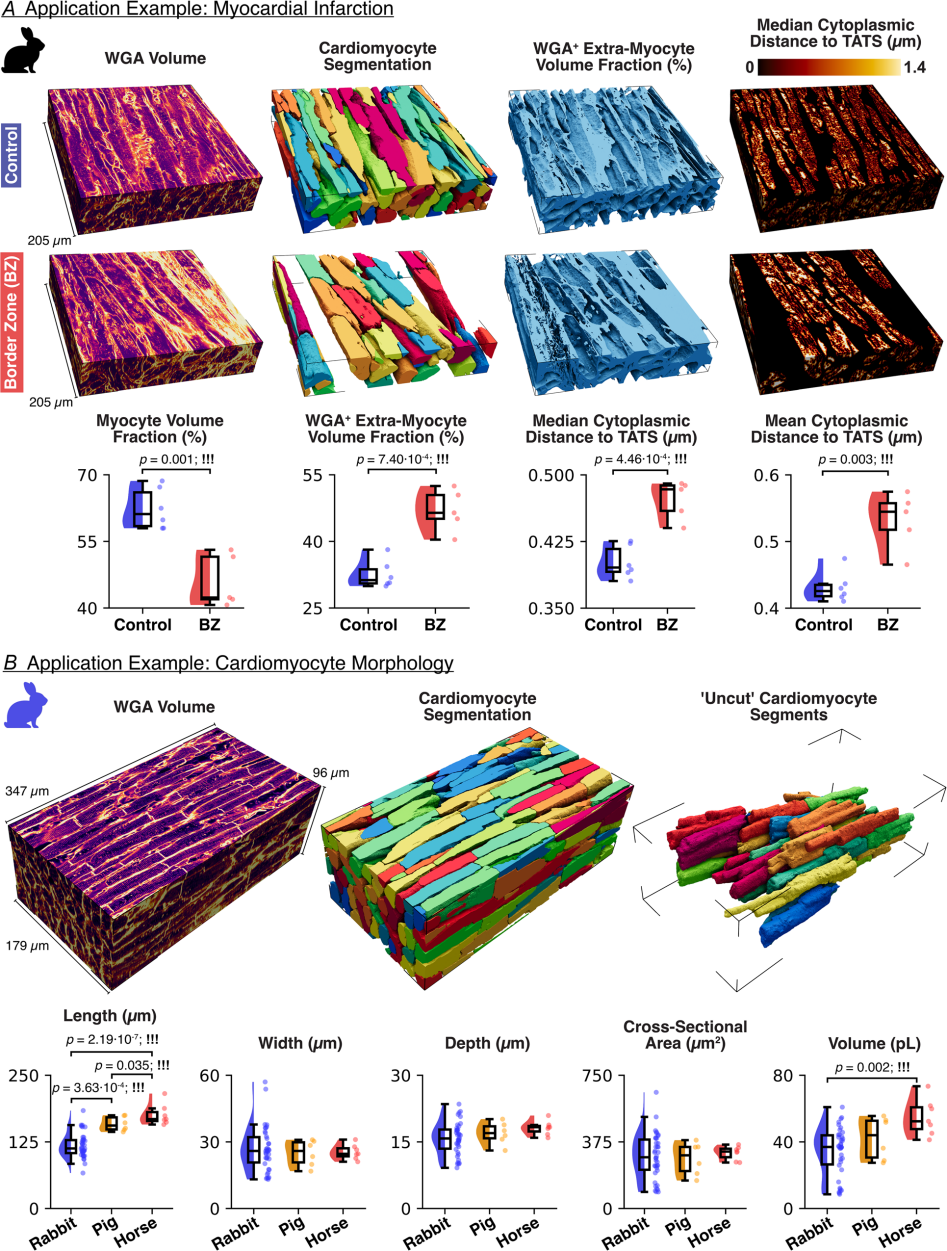
Example analyses utilising cardiomyocyte segmentation *A*, analyses of microstructural tissue composition (myocyte and WGA‐positive extra‐myocyte volume fractions) and subcellular structures (mean/median distance of the cytoplasm to the nearest transverse‐axial tubular system voxel; TATS) within the context of cardiac (patho‐)physiology. *n* = 6/5 image volumes for control and border zone, respectively. Effect sizes are indicated by ‘!’, ‘!!’ and ‘!!!’ for small, medium, and large effects, respectively. *B*, extensive morphological analyses of cardiomyocytes using proofread automated segmentations. Only cardiomyocytes that are not intersecting with the image volume boundaries were used for quantifications. *n* = 42, 7 and 6 cells for rabbit, horse, and pig, respectively.

Here, we demonstrate two downstream applications. First, we quantified cellular composition and subcellular structures in the context of myocardial infarction (Fig. 6*A*; dataset I, proofread segmentations). Compared to control tissue, we observed a smaller cardiomyocyte volume fraction (45.94 % *vs*. 62.36 %, *P* = 1.2 × 10^−3^, *d* = 3.1; *n* = 6/5 image volumes for control and border zone, respectively) and a higher volume fraction of WGA‐positive extra‐myocyte volume (32.58 % *vs*. 46.97 %, *P* = 7.4 × 10^−4^, *d* = 3.7) in the infarct border zone. Additionally, the mean and median distances of the cytoplasm to the nearest TATS voxel were higher in the infarct border zone compared to control tissue (0.53 µm *vs*. 0.43 µm, *P* = 3.4 × 10^−3^, *d *= 3.0 and 0.47 µm *vs*. 0.40 µm, *P* = 4.5 × 10^−4^, *d* = 3.5, respectively).

**Figure A3:**
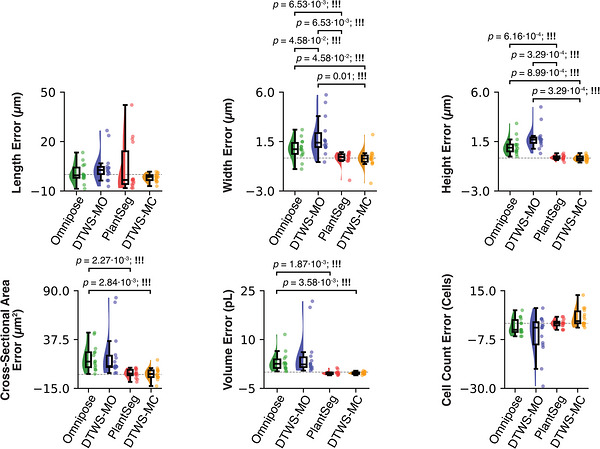
Errors of automated cardiomyocyte segmentation strategies in a morphological analysis The dashed lines represent an error of 0. Small, medium, and large effect sizes are indicated by ‘!’, ‘!!’ and ‘!!!’, respectively.

Second, we conducted a morphological analysis of cardiomyocytes in the context of an interspecies comparison (Fig. 6*B*, dataset II, proofread segmentations). We analysed cardiomyocytes that were not intersecting with the image volume boundaries and extracted their length, width, depth, cross‐sectional area and volume. Only image volumes for rabbit, pig and horse myocardium were large enough to contain high enough numbers of complete cardiomyocytes; thus, the comparison was focused on these species. Cardiomyocyte length was smaller in rabbit (118.11 µm, *n* = 42 cells) compared to pig (158.66 µm, *P* = 3.6 × 10^−4^, *d* = 1.8; *n* = 7 cells) and horse (175.86 µm, *P* = 2.2 × 10^−7^, *d* = 2.5, *n* = 6 cells). Cardiomyocyte volume was substantially lower in rabbit compared to horse (34.28 pL *vs*. 54.85 pL, *P* = 0.002, *d* = 1.5). To generate a lower‐bound estimate of morphological features in volumes where most cardiomyocytes were truncated by the image volume boundaries, we conducted a second analysis (Table 4, Table A1). Here, we included the 30 largest cardiomyocytes per stack, even if they intersected with the image volume boundaries, and contrasted our findings with studies of cardiomyocyte morphology in mouse, rat, rabbit and pig (Chen et al., 2007; Natali et al., 2002; Satoh et al., 1996; Scott et al., 2022; Struckman et al., 2023). In addition to analysing using proofread segmentations, we also evaluated the error of our automated segmentation methods without proofreading (see Appendix Fig. A3 and Table A3).

**Table 4:** Morphological properties of cardiomyocytes (mean ± SD).

Species	Study/Dataset	n	Length (µm)	Width (µm)	Depth (µm)	Volume (pL)	CSA (µm^2^)
**Mouse**	*Struckman*	150	127.3^a^	31.4^a^	11.4^a^	19.4^a^	
	Dataset II_2_	90	107.1 ± 25.4	16.1 ± 2.8	10.8 ± 1.7	11.9 ± 40.0	114.2 ± 28.2
**Rat**	*Satoh*	21	141.9 ± 14.9	32.0 ± 4.8	13.3 ± 1.6	34.4 ± 7.0	
	*Natali: EPI S*	240	121.3 ± 1.0^b^	20.1± 0.2^b^	13.9 ± 0.1^b^	18.6 ± 0.4^b^	
	*Chen: Apex*		137.0 ± 6.7	30.6 ± 2.1	12.0 ± 1.5	39.0 ± 3.5	286 ± 32
	Dataset II_2_	150	124.3 ± 26.3	26.9 ± 5.8	16.3 ± 3.1	33.7 ± 11.0	279.0 ± 88.1
**Rabbit**	*Satoh*	14	123.8 ± 14.4	33.6 ± 6.7	12.8 ± 1.4	30.9 ± 5.2	
	Dataset II_1_	38	118.1 ± 23.3	26.7 ± 10.1	15.8 ± 3.6	34.3 ± 14.5	292.2 ± 128.7
	Dataset II_2_	60	120.7 ± 17.2	27.6 ± 9.3	15.3 ± 3.5	33.8 ± 14.9	284.5 ± 130.2
**Pig**	*Scott*		156.5^a^	24.5^a^	17.5^a^		
	Dataset II_1_	6	158.7 ± 13.6	25.0 ± 5.8	16.9 ± 2.5	42.1 ± 12.8	280.7 ± 92.1
	Dataset II_2_	210	168.7 ± 31.5	31.6 ± 6.9	18.1 ± 3.1	54.7 ± 12.9	379.2 ± 98.5
**Horse**	Dataset II_1_	7	175.9 ± 20.1	25.4 ± 3.3	18.1 ± 1.6	54.9 ± 11.0	312.8 ± 38.7
	Dataset II_2_	180	162.7 ± 45.8	28.8 ± 6.3	17.6 ± 3.8	50.1 ± 17.7	321.9 ± 104.6
**Elephant**	Dataset II_2_	22	111.5 ± 52.2	24.1 ± 10.6	13.6 ± 5.0	29.0 ± 26.2	215.5 ± 138.4

*Note*: Dataset II_1_: Only cells that do not intersect with the image volume boundaries were analysed. Dataset II_2_: Top 30 cells by volume analysed per stack.

Abbreviation: CSA, cross‐sectional area.

^a^ Median value. ^b^ Mean ± SEM.

## Discussion

The 3D microstructure of the myocardium is an important determinant of physiological function in the healthy and pathologically remodelled heart, making it a highly relevant source of information for cardiac research. The present study introduces a comprehensive and accessible toolkit for reconstructing cardiac microstructure from confocal microscopy volumes. Our toolkit includes a reliable sample preparation protocol and a flexible segmentation approach that can be adapted to new data. Importantly, our toolkit and datasets are open‐source and publicly available.

We provide a GUI to run our best‐performing workflow, which includes image restoration and segmentation. Additionally, we provide SegmentPuzzler executables for Windows, Linux and macOS, aiding easy setup. Finally, we expect that our aggregated datasets will have utility for researchers building machine learning and computational models, and for those studying cardiac microstructure.

Our sample preparation method builds upon our previous work (Seidel et al., 2016), with several improvements, including longer staining times for improved penetration of labelling reagents and adjusted humidity control. Our sample preparation method was robust across multiple species and experimental conditions, quick to complete in just a few days, compression‐free and cost‐effective with approximately €5 material cost per stained tissue slice. It consistently enabled high‐quality boundary estimation of cardiomyocytes. Using this protocol and our image restoration method, we achieved imaging depths of up to 240 µm, which is notable for a sample preparation protocol that relies on passive refractive index matching without active clearing. For the acquisition of new image volumes, we recommend an isotropic voxel size of 200 nm to match our training data closely. Thus, a standard confocal or two‐photon microscope should suffice; no specialised hardware is necessary.

We acknowledge several limitations regarding our dataset and imaging workflow: although the maximal depth reached was 240 µm, the typical depth reachable was considerably lower and usually close to 100 µm. Nevertheless, this generally included more than one layer of cardiomyocytes, which typically have an average lateral dimension of less than 40 µm across the species assessed. This imaging depth distinctly exceeds previously reported volume depths for 3D cardiomyocyte segmentation. An additional limitation comes from the fact that WGA does not bind to the cardiomyocyte membranes directly; instead, it binds to components of the glycocalyx on the cell surface. This indirect membrane labelling can lead to ambiguities; for example, where a cardiomyocyte membrane may be present, but not outlined by WGA. Of note, we frequently observed WGA staining in perinuclear regions, consistent with the location of the Golgi apparatus; however, without additional staining, we can't confirm the identity of these structures with certainty. Dataset II relies exclusively on post‐mortem tissue, often obtained from animals killed for other experimental studies or from animals that died of natural causes, thereby maximising tissue utilisation and supporting the 3R principle. As an *ex vivo* method development study, only a subset of the ARRIVE guidelines, which are intended for *in vivo* experiments, was applicable to our study (e.g. no intervention groups, randomisation, blinding, etc.). Additionally, we could not establish the precise age, weight and sex of all animals, which we acknowledge as a limitation of our dataset.

Lastly, our annotations do not precisely follow the finer details of the sarcolemma but focus on overall cardiomyocyte structure. As a result of the large image volumes involved, we proofread pre‐merged volumes instead of creating them from scratch, which carries the risk of overlooking minor errors that may induce bias. Employing more advanced segmentation strategies, such as integrating an edge classifier or biological priors into the MultiCut workflow (Berg et al., 2019; Pape et al., 2019), or incorporating optimised agglomeration strategies (Bailoni et al., 2022; Januszewski et al., 2018), may further reduce merge errors.

Our image restoration workflow significantly reduced spatially varying blur and noise in our datasets, and we consider that it has potential applications beyond image segmentation, including other imaging modalities and datasets. The model with a data‐driven architecture based on CARE performed better than an image‐formation‐driven architecture based on LUCYD. However, we note that LUCYD was initially designed to restore deconvolved images, which is a slightly different task from what we test here. Although we have not assessed whether automatic segmentation methods benefit from image restoration, working with denoised images has substantially eased semi‐automatic proofreading. Furthermore, image restoration may encourage researchers to trade slow imaging speed for larger acquired volumes and less photobleaching because some of the lost image quality may be recoverable during restoration. Although the methodology is not novel (Chobola et al., 2023; Li et al., 2022; Weigert et al., 2018), our approach of using varying pinhole sizes to generate degraded and clean images to train machine learning algorithms has not been used before.

Furthermore, we set up and assessed the performance of algorithms for 3D segmentation of cardiomyocytes on large and diverse datasets, spanning species of varying size, from mice to humans and beyond. Datasets include a disease model (myocardial infarction) as well as increasingly popular cardiac tissue culture models. We identified DTWS‐MC as the best‐performing approach. In our pipeline, a semantic cardiomyocyte mask is used to reduce merge errors with the extra‐myocyte space. Future work may also introduce further semantic masks; for example, to distinguish Purkinje or pacemaker cells, adding further granularity to the cardiomyocyte segmentation. DTWS‐MC works particularly well with SegmentPuzzler because it has a higher split error, relative to its merge error, which can be easily corrected using our tool. We are not aware of other automatic deep learning‐based methods that were applied to microstructural 3D reconstruction of cardiomyocytes. Although methods for automated 2D segmentation of cardiomyocytes exist (da Silva et al., 2022; Droste et al., 2023), scaling these to 3D is challenging, in particular for large datasets. As a result, manual and semi‐automated methods are used extensively for reconstructions in 3D (Bensley et al., 2016; Dileep et al., 2023; Greiner et al., 2018, 2022; Seidel et al., 2016). These methods are typically very time‐consuming, with estimates spanning from 300–400 cells per day (Bensley et al., 2016) to 2–3 days per 204 × 204 × 60 µm image volume (Seidel et al., 2016). We successfully demonstrate the applicability of our workflow to two stacks of previously unseen conditions and species (humans), which were notably different from the training data. Additionally, one stack featured a highly unusual change in cardiomyocyte orientation. On average, proofreading took ∼1 h per stack (205 × 205 × 54 µm image volume) or 20 s per cell fragment, allowing reconstruction of 1200 cells per 8 h workday. It is worth noting that this represents a worst‐case scenario; we expect that proofreading times can be significantly shorter with additional training on data from similar conditions.

The decision to proofread using SegmentPuzzler may depend on the specific research goals associated with the segmentation task. However, with the algorithms presented here, we strongly encourage routine visual inspection of resulting segmentations. If the goal is to assess individual cardiomyocyte morphological properties, such as to evaluate cellular hypertrophy, the output of our automated method may be sufficient without proofreading because population statistics will probably remain representative (see Appendix, Fig. A3 and Table A3). Critically, compared to 2D analyses of cardiomyocyte morphology, 3D analysis provides more dependable insight (such as regarding cell cross‐section, or TATS spacing; e.g. as an indication of sarcomere length). However, if the goal is to reconstruct a specific volume (e.g. for computational models of various constituents), we recommend additional proofreading in SegmentPuzzler. If multiple stacks require substantial manual correction, we recommend iterations of partially annotating stacks and retraining the model. Of note, we expect the segmentation workflows to generalise to different segmentation tasks and image modalities, as long as strong boundary features of biological structures are discernible at the imaging data resolution. We found that the models improve quickly using the outlined human‐in‐the‐loop approach. The resulting realistic 3D cardiomyocyte geometries, now available as an open resource (see Data availability statement below), may serve as a key input as training data for current and future cell reconstruction algorithms, as well as for computational models of microscale electrophysiology and electromechanics, offering an alternative to commonly used synthetic approximations (Jæger et al., 2021; Steyer et al., 2023; Telle et al., 2023).

We demonstrated two of many potential downstream applications of cardiomyocyte segmentation. First, we measured the distance from the cardiomyocyte cytoplasm to the nearest TATS in a rabbit model of myocardial infarction and observed a higher average distance in the scar border zone compared to sham‐operated controls, consistent with reported post‐myocardial infarction remodelling (Heinzel et al., 2008; Kemi et al., 2011). Interestingly, despite differences in species and disease aetiology, the median distances for rabbit myocardial infarction border zone and sham tissue (0.47 µm *vs*. 0.40 µm, a 0.07 µm difference) are remarkably close to median distances reported for a sheep tachypacing heart failure model (0.45 µm *vs*. 0.38 µm; also a 0.07 µm difference; Caldwell et al., 2014). Second, we investigated the cardiomyocyte morphology across species, also largely in keeping with reported literature values originating from 3D analyses (Chen et al., 2007; Natali et al., 2002; Satoh et al., 1996; Scott et al., 2022; Struckman et al., 2023). However, future in‐depth analyses would probably benefit from a larger, standardised dataset, ideally one that matches age, sex and sampling location across different species, and applies a uniform protocol for tissue sampling and processing. Harmonisation of those variables, combined with an unbiased, automated analysis such as ours, should help to clearly separate biological differences from methodological ones.

In summary, we introduce an open and accessible deep learning‐enabled toolkit for reconstructing ventricular cardiomyocytes, providing a large and diverse dataset across various species and experimental conditions. We anticipate our toolkit to facilitate quantitative characterisation based on realistic 3D cardiomyocyte morphology, ultimately advancing our understanding of cardiac (patho‐)physiology.

## Additional information

## Competing interests

The authors declare that they have no competing interests.

## Author contributions

J.G. was responsible for conceptualisation and methodology, developed software, performed formal analysis, curated data, wrote the original draft, edited the manuscript, and carried out visualisation. F.F. was responsible for methodology (DTWS‐MO), developed software (DTWS‐MO), and reviewed and edited the paper. F.S. curated data and reviewed and edited the paper. J.M. was responsible for data curation, and reviewed and edited the paper. T.S. was responsible for conceptualisation, methodology, curated data, reviewed and edited the paper, and acquired funding. P.K. was responsible for conceptualisation, reviewed and edited the paper, and acquired funding. E.A.R.‐Z. was responsible for conceptualisation, curated data, reviewed and edited the paper, acquired funding, and supervised the study. All authors approved the final version of the manuscript submitted for publication and agree to be accountable for all aspects of the work in ensuring that questions related to the accuracy or integrity of any part of the work are appropriately investigated and resolved. All persons designated as authors qualify for authorship, and all those who qualify for authorship are listed.

## Funding

This work was funded by the German Research Foundation (DFG) through an Emmy Noether Fellowship (#396913060 to E. A. Rog‐Zielinska), the collaborative research centre CRC1425 (#422681845) and the Cluster of Excellence CIBSS CEXC‐2189 (#390939984 via E. A. Rog‐Zielinska and P. Kohl), as well as project support (#505991664 to T. Seidel). Imaging data were partly recorded at the SCI‐MED imaging facility with an instrument funded by the DFG (#404198760 to P. Kohl).

## Supporting information


Peer Review History

## Data Availability

Data and code repositories are publicly available. Restoration and agglomeration algorithms, including a GUI, are available at https://github.com/JoeGreiner/CMSegmentationToolkit. SegmentPuzzler, our semi‐automatic segmentation tool for annotation and proofreading, including executables/AppImages for Windows, macOS and Linux, is available at https://github.com/JoeGreiner/SegmentPuzzler. The software used to create the supplementary web visualisations, BokehBioImageDataVis, is available at https://github.com/JoeGreiner/BokehBioImageDataVis. Image data, corresponding cardiomyocyte annotations, and supplementary web visualisations are available at https://doi.org/10.5281/zenodo.14390418.

## Appendix

**Table A1:** Glossary of key image segmentation terminology.

Terminology	Lay description
**Segmentation**	Process of partitioning an image into meaningful segments. Cardiomyocyte segmentation in the context of the paper is the process of ‘extracting’ the shape of cardiomyocytes from 3D microscopy volumes. Importantly, in this manuscript, we are performing instance segmentation, where we not only decide if parts of the volume are cardiomyocyte or not, but also we aim to separate individual instances of cardiomyocytes.
**Pixel**	Short for ‘picture element’. Smallest addressable element in a 2D raster image.
**Voxel**	Short for ‘volume element’. The 3D analogue of a pixel.
**Superpixel/supervoxel**	Meaningful groupings of pixels/voxels, based on similarity of boundaries, textures, or other features. The number of groupings is typically much less than the number of pixels. For example, in a 512 × 512 pixels image (262,144 pixels), working with 200 superpixels is probably much faster and often more robust than processing every pixel. This benefit is even more pronounced in higher dimensions, such as 3D volumes.
**Watershed transform**	An algorithm that groups pixels or voxels. Metaphorically, image/volume intensity values are interpreted as heights of a topographic map, and groupings of pixels/voxels are created by filling valleys with ‘water’, thus mapping the ridges.
**Distance transform**	Image processing technique where each pixel/voxel is replaced by the distance to a given structure. For example, the distance transform of the nucleus would indicate for each pixel/voxel the distance to the nearest nucleus surface element.
**Distance‐transform watershed**	Specialisation of the watershed algorithm. In the context of the paper, we use a distance‐transform watershed of cardiomyocyte boundary predictions. In other words, we use deep learning to predict the boundaries of cardiomyocytes in microscopy images, and then create a new image that indicates the distance of each pixel to the nearest boundary. This distancemap is inverted, so that predicted boundaries become ridges, and the watershed creates groupings of voxels that follow these ridges. Importantly, the supervoxels are able to ‘close holes’ in the predicted boundaries, as they naturally extend predicted boundaries.
**Adapted rand error**	Common metric to evaluate instance segmentations, where all unordered pairs of voxels in the prediction/ground truth are evaluated with a simple yes/no question: Are these two voxels in the same object in the prediction? Are they in the same object in the ground truth? In doing so, each pair of voxels can be classified as either true positive (TP, part of the same object in both), true negative (TN, part of different objects in both), false negative (FN, a prediction that would erroneously split an object), or false positive (FP, a prediction that would erroneously merge two objects). An Adapted Rand Error of 0 indicates complete agreement between segmentations, while a value of 1 indicates complete disagreement.
**Split error**	The split error measures how much more information is needed to describe the predicted labels once one knows the ground truth labels. It captures over‐segmentation, that is one cardiomyocyte split into multiple fragments.
**Merge error**	The merge error measures how much more information is needed to describe the ground truth labels once one knows the predicted labels. It captures under‐segmentation, that is multiple cardiomyocytes are (falsely) merged together.

**Table A2:** Segmentation run time (∼800 million voxel volume).

Method	Time (min)	Adapted Rand Error
Omnipose	**3.92**	0.11
PlantSeg	14.60	0.10
DTWS‐MC	60.82	**0.06**
DTWS‐MC‐no‐TTA	16.73	0.07

*Note*: Best observed metric is indicated in bold. TTA: test‐time augmentation. Testing was conducted on a computer equipped with an Intel i9‐13900K processor, 128 GB of RAM and an Nvidia RTX 4090 graphics card. We report the average from three runs. Timing was recorded on an image stack with 2778 × 1016 × 280 voxels, representing a larger‐than‐average image stack.

**Table A3:** Morphological properties of cardiomyocytes (test set; mean ± SD).

Method	Cell count (Cells)	Length (µm)	Width (µm)	Height (µm)	Volume (pL)	CSA (µm^2^)
Manual proofreading	70.07 ± 50.60	109.89 ± 35.51	22.27 ± 6.36	11.22 ± 5.35	23.69 ± 18.20	181.22 ± 124.72
Omnipose	68.71 ± 51.17	110.66 ± 35.96	23.09 ± 6.72	12.33 ± 5.45	26.82 ± 20.61	199.09 ± 136.30
DTWS‐MO	64.36 ± 47.09	114.42 ± 39.34	24.22 ± 7.60	13.06 ± 5.85	28.73 ± 22.55	202.68 ± 142.02
PlantSeg	69.93 ± 50.33	113.87 ± 45.57	22.24 ± 6.32	11.26 ± 5.30	23.48 ± 18.43	182.14 ± 123.91
DTWS‐MC	72.79 ± 53.19	107.99 ± 36.49	22.24 ± 6.26	11.19 ± 5.25	23.40 ± 18.22	182.09 ± 123.58

Abbreviation: CSA, cross‐sectional area.
