# Supplementary material for: A deep learning‐enabled toolkit for the 3D segmentation of ventricular cardiomyocytes
Source: J Physiol. 2025 Oct 1;604(13):5561–84. doi: 10.1113/JP288557 (PMC13327770; doi:10.1113/JP288557)
Supplement: Supplementary file 1 — Peer Review History [file TJP-604-5561-s001.pdf]

The Journal of Physiology

A Deep Learning-Enabled Toolkit for the 3D Segmentation of Ventricular Cardiomyocytes

Joachim Greiner, Fabio Frangiamore, Frédéric Sonak, Josef Madl, Thomas Seidel, Peter Kohl, and Eva A. Rog-Zielinska
DOI: 10.1113/JP288557

Corresponding author(s): Joachim Greiner (joachim.greiner@uniklinik-freiburg.de)

The following individual(s) involved in review of this submission have agreed to reveal their identity: Ken D O'Halloran (Referee #3)

Review Timeline:

Submission Date:	17-Mar-2025
Editorial Decision:	22-May-2025
Revision Received:	22-Jul-2025
Editorial Decision:	11-Aug-2025
Revision Received:	14-Aug-2025
Accepted:	18-Aug-2025

Senior Editor: Bjorn Knollmann

Reviewing Editor: Eleonora Grandi

Transaction Report:

(Note: With the exception of the correction of typographical or spelling errors that could be a source of ambiguity, letters and reports are not edited. Depending on transfer agreements, referee reports obtained elsewhere may or may not be included in this compilation. Referee reports are anonymous unless the Referee chooses to sign their reports.)

Dear Dr Greiner,

Re: JP-RP-2025-288557 "A Deep Learning-Enabled Toolkit for the 3D Segmentation of Ventricular Cardiomyocytes" by Joachim Greiner, Fabio Frangiamore, Frédéric Sonak, Josef Madl, Thomas Seidel, Peter Kohl, and Eva A. Rog-Zielinska

Thank you for submitting your manuscript to The Journal of Physiology. It has been assessed by a Reviewing Editor and by 2 expert referees and we are pleased to tell you that it is potentially acceptable for publication following satisfactory major revision.

Please advise your co-authors of this decision as soon as possible.

The referee reports are copied at the end of this email.

Please address all the points raised and incorporate all requested revisions or explain in your Response to Referees why a change has not been made. We hope you will find the comments helpful and that you will be able to return your revised manuscript within 2 months. If you require longer than this, please contact journal staff: jp@physoc.org. Please note that this letter does not constitute a guarantee for acceptance of your revised manuscript.

Your revised manuscript should be submitted online using the link in your Author Tasks: Link Not Available. This link is accessible via your account as Corresponding Author; it is not available to your co-authors. If this presents a problem, please contact journal staff (jp@physoc.org). Image files from the previous version are retained on the system. Please ensure you replace or remove any files that are being revised.

If you do not wish to submit a revised version of your manuscript, you must inform our journal staff (jp@physoc.org) or reply to this email to request withdrawal. Please note that a manuscript must be formally withdrawn from the peer review process at one journal before it may be submitted to another journal.

TRANSPARENT PEER REVIEW POLICY: To improve the transparency of its peer review process, The Journal of Physiology publishes online as supporting information the peer review history of all articles accepted for publication. Readers will have access to decision letters, including Editors' comments and referee reports, for each version of the manuscript, as well as any author responses to peer review comments. Referees can decide whether or not they wish to be named on the peer review history document.

ABSTRACT FIGURES: Authors are expected to use The Journal's premium BioRender account to create/redraw their Abstract Figures. Information on how to access this account is here: https://physoc.onlinelibrary.wiley.com/journal/14697793/biorender-access.

This will enable Authors to create and download high-resolution figures. If authors have used the free BioRender service, they can use the instructions provided in the link above to download a high-resolution version suitable for publication.

The link provided should only be used for the purposes of this submission. Authors will be charged for figures created on this account if they are not related to this manuscript submission.

LANGUAGE EDITING AND SUPPORT FOR PUBLICATION: If you would like help with English language editing, or other article preparation support, Wiley Editing Services offers expert help, including English Language Editing, as well as translation, manuscript formatting, and figure formatting at www.wileyauthors.com/eeo/preparation. You can also find resources for Preparing Your Article for general guidance about writing and preparing your manuscript at www.wileyauthors.com/eeo/prepresources.

RIGOUR AND REPRODUCIBILITY: We recommend authors consult The Journal's Rigour and Reproducibility requirements to ensure that their manuscript is suitable for publication: https://physoc.onlinelibrary.wiley.com/pb-assets/hub-assets/physoc/documents/TJP-Rigour-and-Reproducibility-Requirements.pdf

REVISION CHECKLIST:

Check that your Methods section conforms to journal policy: https://jp.msubmit.net/cgi-bin/main.plex?form_type=display_requirements#methods.

Check that data presented conforms to the statistics policy: https://jp.msubmit.net/cgi-bin/main.plex?form_type=display_requirements#statistics.

Upload a full Response to Referees file. To create your 'Response to Referees': copy all the reports, including any comments from the Senior and Reviewing Editors, into a Microsoft Word, or similar, file and respond to each point, using font or background colour to distinguish comments and responses and upload as the required file type.

Please upload two versions of your manuscript text: one with all relevant changes highlighted and one clean version with no

changes tracked. The manuscript file should include all tables and figure legends, but each figure/graph should be uploaded as separate, high-resolution files.

You may also upload:

- 'Potential Cover Art' for consideration as the issue's cover image
- Appropriate Supporting Information (Video, audio or data set: see https://jp.msubmit.net/cgi-bin/main.plex?form_type=display_requirements#supp).

We look forward to receiving your revised submission.

If you have any queries, please reply to this email and we will be pleased to advise.

Yours sincerely,

Bjorn Knollmann
Senior Editor
The Journal of Physiology

REQUIRED ITEMS

- Author photo and profile. First or joint first authors are asked to provide a short biography (no more than 100 words for one author or 150 words in total for joint first authors) and a portrait photograph. These should be uploaded and clearly labelled together in a Word document with the revised version of the manuscript. See Information for Authors for further details.

- You must start the Methods section with a paragraph headed Ethical approval (https://jp.msubmit.net/cgi-bin/main.plex?form_type=display_requirements#methods).

Research must comply with The Journal's policies regarding animal experiments (https://physoc.onlinelibrary.wiley.com/hub/animal-experiments) and adherence to these policies must be stated in the manuscript.

Authors should confirm in their Methods section that their experiments were carried out according to the guidelines laid down by their institution's animal welfare committee, including an ethics approval reference number. The Methods section must contain a statement about access to food, water and housing, details of the anaesthetic regime: anaesthetic used, dose and route of administration, and method of killing the experimental animals.

- Please upload separate high-quality figure files via the submission form.

- Please include an Abstract Figure file, as well as the Figure Legend text within the main article file. The Abstract Figure is a piece of artwork designed to give readers an immediate understanding of the research and should summarise the main conclusions. If possible, the image should be easily 'readable' from left to right or top to bottom. It should show the physiological relevance of the manuscript so readers can assess the importance and content of its findings. Abstract Figures should not merely recapitulate other figures in the manuscript. Please try to keep the diagram as simple as possible and without superfluous information that may distract from the main conclusion(s). Abstract Figures must be provided by authors no later than the revised manuscript stage and should be uploaded as a separate file during online submission labelled as File Type 'Abstract Figure'. Please also ensure that you include the figure legend in the main article file. All Abstract Figures should be created using BioRender. Authors should use The Journal's premium BioRender account to export high-resolution images. Details on how to use and access the premium account are included as part of this email.

EDITOR COMMENTS

Reviewing Editor:

Ethics Concerns:
The authors use tissue from their own institution as well as the Universities of Utah and Erlangen. Human datasets were

obtained with ethical approval from the respective institutions. Much animal data is from the authors' own institution and therefore more detail is required to fulfill the ARRIVE guidelines.

First, my apologies for the extreme delay in reviewing this manuscript.

The reviewers find the manuscript an tool presented potentially very impactful, but would like to see it revised to (1) make the language accessible to non imaging experts; and (2) include examples, testing (e.g., comparison with manual methods), and application to the real world (what questions in physiology can this tool allow addressing?).

Please also see 'Required Items' above.

Senior Editor:

I concur with the reviewing editor's recommendation. Please comply to the journal's ethics policy.

REFEREE COMMENTS

Referee #1:

The authors present a deep learning enabled toolkit for the segmentation of cardiomyocytes. The method was applied to confocal microscopy data from 7 different species. The authors demonstrate that the CARE model was best for image restoration, and on these restored images, a low Rand error was achieved for automatic segmentations with the DTWS-MC pipeline. The authors conclude that this approach for 3D cardiomyocyte segmentation will improve our understanding of cardiac (patho-)physiology. Please find my comments regarding this study below.

General comments:

1. The statements made on lines 26-28 and lines 479-481 are a bit unclear to me. The manuscript is very technical with few real-world examples for how their new approach will specifically enhance our knowledge of 3D cardiomyocytes with respect to cardiac (patho-)physiology. For example, what was the question driving the need for this technology? What level of precision, both in time and space, does the new approach by the authors need to achieve? The take home message to non-imaging communities could be refined in my opinion.

2. Do the authors think that their method can be applied to other high-resolution imaging modalities such as micro-CT or OCT?

3. Can the algorithm differentiate between multiple cell types in a volume of tissue based on shape? For example, Purkinje cells versus ventricular myocytes in the ventricular myocardium?

Specific comments:

Line 47: I had trouble finding the cells/minute results in the manuscript.

Line 79-92: Have the authors directly compared their new approach with the benchmarks of the previous approaches on

lines 79-92 in the introduction.

Line 100: "SegmentPuzzler", is this separate from the automatic segmentations mentioned earlier in the abstract? I imagine the methods will explain this, but to this point it is unclear to me the difference/reason for doing both automated and semi-automated segementation techniques. Is SegmentPuzzler basically a user-friendly interface to perform and/or visualize the segmentation of cardiomyocytes?

Line 103: Is the major flaw of the previous approaches listed in the introduction the input data (quality, resolution, preparation, etc.) or from shortcomings in the algorithms used in their methods? For example, would past methods also work better with higher quality data, as the authors appear to do with their image restoration method? I imagine there is also a balance between processing time and accuracy.

Line 119: Is each data set number I,II, and III linked to the data in Table 1?

Lines 169, 174, 189: There are different out-of-plane and voxel resolutions between the different datasets. What is the minimal resolution needed before the shape of the cells becomes distorted? This should likely be determined for the smallest cell across the 7 species.

Line 219: What are the units of 101? Also, why 101?

Line 222: Again, what are the units of 150?

Line 331: For the various datasets, is it known how many cardiacmyocytes are present on average in each species for 1um^3 of tissue?

Line 349: In addition to accuracy, I imagine that the time needed to perform the segmentation is also important. Can the authors comment on the speed of each approached tried with respect to accuracy?

Line 381: The main point of the results in this section are unclear to me.

Line 404: The authors mention that their method and/or datasets could improve computational models. How so specifically?

Line 457: I think some of these time benchmarks could be moved to the results section.

Line 464: Could SegmentPuzzler improve the diagnosis/treatment of cardiac abnormalities associated with cellular hypertrophy? If so, what specific type of information would be transferred to a clinician or their technicians?

Referee #2:

In this manuscript the authors present a freely available toolkit for the automatic segmentation of 3-D confocal Z stacks from cardiomyocytes. I agreed with the authors that a 3-D view of the myocardium is extremely useful for understanding cardiac pathophysiology and that analytical toolkits are currently insufficient. Researchers often rely on manual segmentation which is not time or cost-effective. Therefore, new ways to understand the 3-D architecture of cardiac tissue would be welcomed in the field. I would have thought therefore that this study would be interesting for those working on the structure of the myocardium.

The methods implemented in the design of the toolkit are not within my area of expertise but the authors do seem to have taken great care with the development. For example, 2 types of 3-D segmentation strategies were used firstly looking at XY slices and secondly using YZ and XZ slices. It's also interesting to see the use of different species, disease states and the inclusion of cultured myocardium for the evaluation.

The manuscript is by necessity technical. However, given its target audience, the authors may wish to increase accessibility generally to help the end user understand how the toolkit works and where it strengths and weaknesses lie. With this in mind it would be helpful to have brief explanations of key terms such as supervoxel, Rand error, split error, merge error and watershed algorithm for the non-specialist reader.

In figure 2, for the cells in the annotations, it would be useful to relate them to the XY image shown above. Maybe this could be achieved with coloured asterisks in the XY image to highlight particular cells to the reader?

In figure 3 the legend is very helpful and takes the reader through the process the authors have employed. However, in the results text this is less clear. Could the authors add more detail to the results text to guide the reader through the data in more detail making reference to the separate panels in figure 3? It would also help in table 3 to make it clear that the merge and split columns refer to errors.

For figure 4 it would also be useful to mention the specific panels in figure for in the results section to help guide the non-specialist reader.

At line 365 the authors state DTWS - MC and PlantSeg produced obvious split errors especially in regions containing branching cardio myocytes. It is not clear to me exactly what the authors are referring to in panel B. Could the author show these errors? Perhaps by zooming in and explaining in the results text? Would this be helpful to potential users so they could spot errors in their own data sets? It would also be useful to see examples of cells which falsely extend into the extra cellular space. Similarly, it would be helpful for the authors to highlight to the reader examples of merge errors.

In section 3.4 the authors have assessed the utility of their method by applying the best performing method to 2 stacks of human myocardium. While the authors discuss this and give their calculated errors, they should also present the data in the form of a figure. It would be useful to discuss what the errors using the best segmentation method means for the data output in a little more detail.

The data in figure 3D is striking and it appears that the tool kit would be very useful for those interested in understanding subcellular structures in 3-Dimensions and in the case of the images presented, the transverse tubule network. Please could the authors include more information about whether this is possible with their toolkit? For example could the toolkit assess transverse tubule density within a cell or are there problems with thresholding? Could the end user expand the utility of the tool kit for data sets of other cellular components e.g. mitochondria?

To further reassure future users of the software, are the authors able to make measurements from their data sets and compare it to measurements using a manual method? This could for example be a count of the number of cells in a given volume, measurements of cell length or width of volume et cetera. If the toolkit can be used for sub-cellular analysis this could include transverse tubule density or how transverse tubule density changes with disease (this could be compared to

published data).

Did the authors have any problems with WGA staining the golgi in their datasets and if so how was this accounted for?

END OF COMMENTS

Point-by-point response to Reviewing and Senior Editor:

Reviewing Editor:
The authors use tissue from their own institution as well as the Universities of Utah and Erlangen. Human datasets were obtained with ethical approval from the respective institutions. Much animal data is from the authors' own institution and therefore more detail is required to fulfill the ARRIVE guidelines.
Senior Editor:
I concur with the reviewing editor's recommendation. Please comply to the journal's ethics policy.

Answer:
We thank the editors for their recommendations. We have revised the manuscript to cover more aspects of animal-related metadata. Our study exclusively relies on *ex vivo* tissue samples obtained from animals *post-mortem,* which were, in many cases, euthanised for other scientific experimental series or died of natural causes, maximising tissue use and supporting the 3R principles of reduction. ARRIVE guidelines focus on designing *in vivo* experiments that involve groups, interventions, randomisation, and predefined outcome measures, which are not applicable to *ex vivo* method development studies, such as ours. We have added more detail on relevant aspects and acknowledge this now explicitly in the Discussion.

Reviewing Editor:
The reviewers find the manuscript an tool presented potentially very impactful, but would like to see it revised to (1) make the language accessible to non imaging experts; and (2) include examples, testing (e.g., comparison with manual methods), and application to the real world (what questions in physiology can this tool allow addressing?).

Answer:
We thank the editor and the reviewers for their constructive feedback. We have revised the manuscript accordingly. Specifically, we addressed

- **(1)** by revising the manuscript and introducing a glossary (new Appendix Table A1) aimed at readers without imaging expertise; and
- **(2)** by adding examples of error types (new manuscript Fig. 1, Appendix Fig. A1), testing (new manuscript Appendix Fig. A3, Appendix Table A3, and verification experiments), and extensive real-world examples (new manuscript Fig. 6).

Point-by-point response to reviewer 1:

We would like to thank Reviewer 1 for a careful and constructive review of our manuscript. Please find a point-by-point answer below.

Reviewer 1:
The authors present a deep learning enabled toolkit for the segmentation of cardiomyocytes. The method was applied to confocal microscopy data from 7 different species. The authors demonstrate that the CARE model was best for image restoration, and on these restored images, a low Rand error was achieved for automatic segmentations with the DTWS-MC pipeline. The authors conclude that this approach for 3D cardiomyocyte segmentation will improve our understanding of cardiac (patho-)physiology.

The statements made on lines 26-28 and lines 479-481 are a bit unclear to me. The manuscript is very technical with few real-world examples for how their new approach will specifically enhance our knowledge of 3D cardiomyocytes with respect to cardiac (patho-)physiology. For example, what was the question driving the need for this technology?

Answer:
Thank you for raising this issue. We agree that real-world examples and direct applications are currently not described with sufficient clarity. The driving motivation for this paper was the lack of an **open, robust, and truly three-dimensional (3D) method** for analysing ventricular myocardium. 3D methods overcome pitfalls related to the more commonly used 2D imaging of tissue sections. 2D imaging of thin sections may give rise to significant errors, of which we illustrate and quantify a major one, the lack of a controlled cutting plane, in Fig. 1 (also new manuscript figure: Fig. 1). We hope that this serves as an intuitive introduction to the challenges of 2D analyses for non-technical readers.

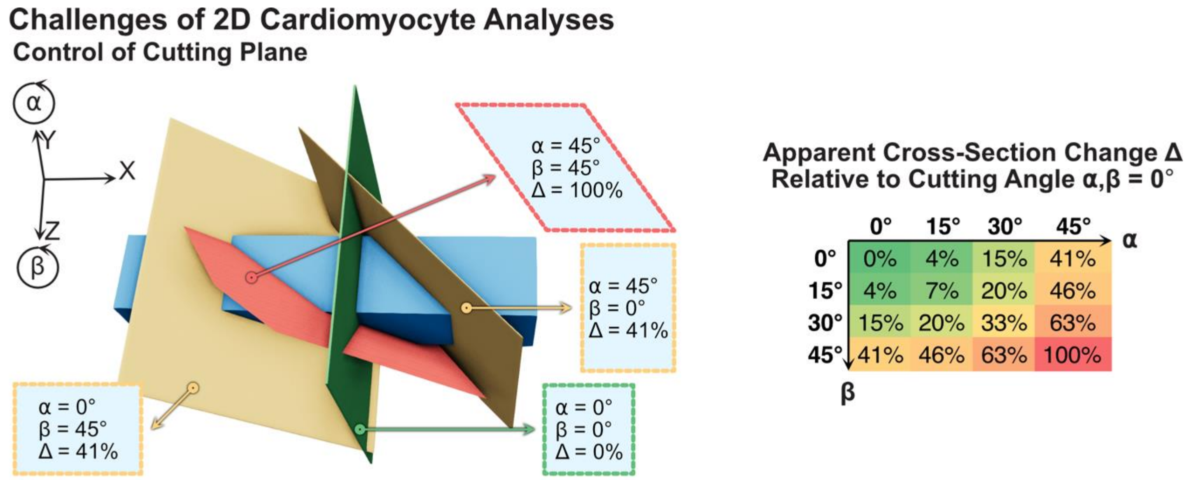

Fig. 1: Challenges of 2D cardiomyocyte analyses, related to limited control over the cutting plane. 2D analyses of cardiomyocytes often assume a perfectly orthogonal cutting plane, relative to one of the principal axes of the cell. Here, with the help of a simple 3D block, we demonstrate the effect of non-orthogonal cuts on the determined apparent cross-sectional area, using a numerical simulation. Of note, orientation of cardiomyocytes in tissue can change quickly over a distance as short as 100 µm, so it is nearly impossible to cut all cardiomyocytes within a given field of view in a uniform manner.

Additionally, the updated text now provides concrete examples of the breadth of new questions that could be addressed using our workflows, and we have added a new figure to highlight specific application examples (Fig. 2, new manuscript Fig. 6). Specific application examples are:

1. **Microstructural composition and fibrosis.** Segmenting cardiomyocytes facilitates downstream analyses. For example, a wheat germ agglutinin (WGA)-based segmentation of capillaries and other non-myocytes is feasible with the addition of further antibodies [**Greiner18**]. Even without the inclusion of additional antibodies, a simple analysis of e.g. the WGA-positive extra-myocyte volume can be a useful measure for fibrotic tissue constituents, as WGA also binds to collagens. We provide sample code and illustrate the application of the analysis of WGA-positive extra-

myocyte space in Fig. 2A. Direct applications could include the quantification of fibrosis or the assessment of changes in capillary density after myocardial infarction.

2. **Extensive morphological analyses.** Automated quantification of key cardiomyocyte properties, such as cardiomyocyte orientation, length, width, height, cross-sectional area, and volume. We provide sample code and highlight this application in Fig. 2B. This could be applied, for example, to the unbiased assessment of hypertrophy or of cardiomyocyte disarray in diseased tissue.
3. **Subcellular protein/organelle quantification and localisation.** Protein (RyR, GJA1, ...) or organelle (transverse-axial tubular system [TATS], mitochondria, ...) localisation can be quantified in relation to the cardiomyocyte morphology for any immunofluorescent label, e.g. with regard to distance-to-sarcolemma, distance-to-transverse axial tubular system, or polar/lateral localisations. We provide sample code for the quantification of cytoplasm-to-nearest-TATS distances, based on WGA staining alone in Fig. 2A. Further applications could include the 3D assessment of gap junction lateralisation after ischemia, or of preferential ion channel clustering within cardiomyocyte membrane sub-populations.
4. **Computational electrophysiology and electromechanics.** Our collaborators in the EU-funded network of excellence MICROCARD, as well as other groups, are already using our high-resolution cardiomyocyte reconstructions to build computational meshes for simulation of electrical conduction and mechanical active forces with micrometre resolution. We have also utilised cardiomyocyte reconstructions in prior work where we determined intra- and extracellular conductivity tensors using image-based models [Greiner18, Greiner22]. We expect broad applicability of our tools and methods to other use cases and conditions.

We have therefore extensively revised the manuscript, including new sub-sections for methods (section 2.8) and results (section 3.5).

References:

[Greiner18] Greiner J, Sankarankutty AC, Seemann G, Seidel T, Sachse FB. Confocal microscopy-based estimation of parameters for computational modeling of electrical conduction in the normal and infarcted heart. Frontiers in Physiology. 2018;9:239.

[Greiner22] Greiner J, Sankarankutty AC, Seidel T, Sachse FB. Confocal microscopy-based estimation of intracellular conductivities in myocardium for modeling of the normal and infarcted heart. Computers in Biology and Medicine. 2022;146:105579.

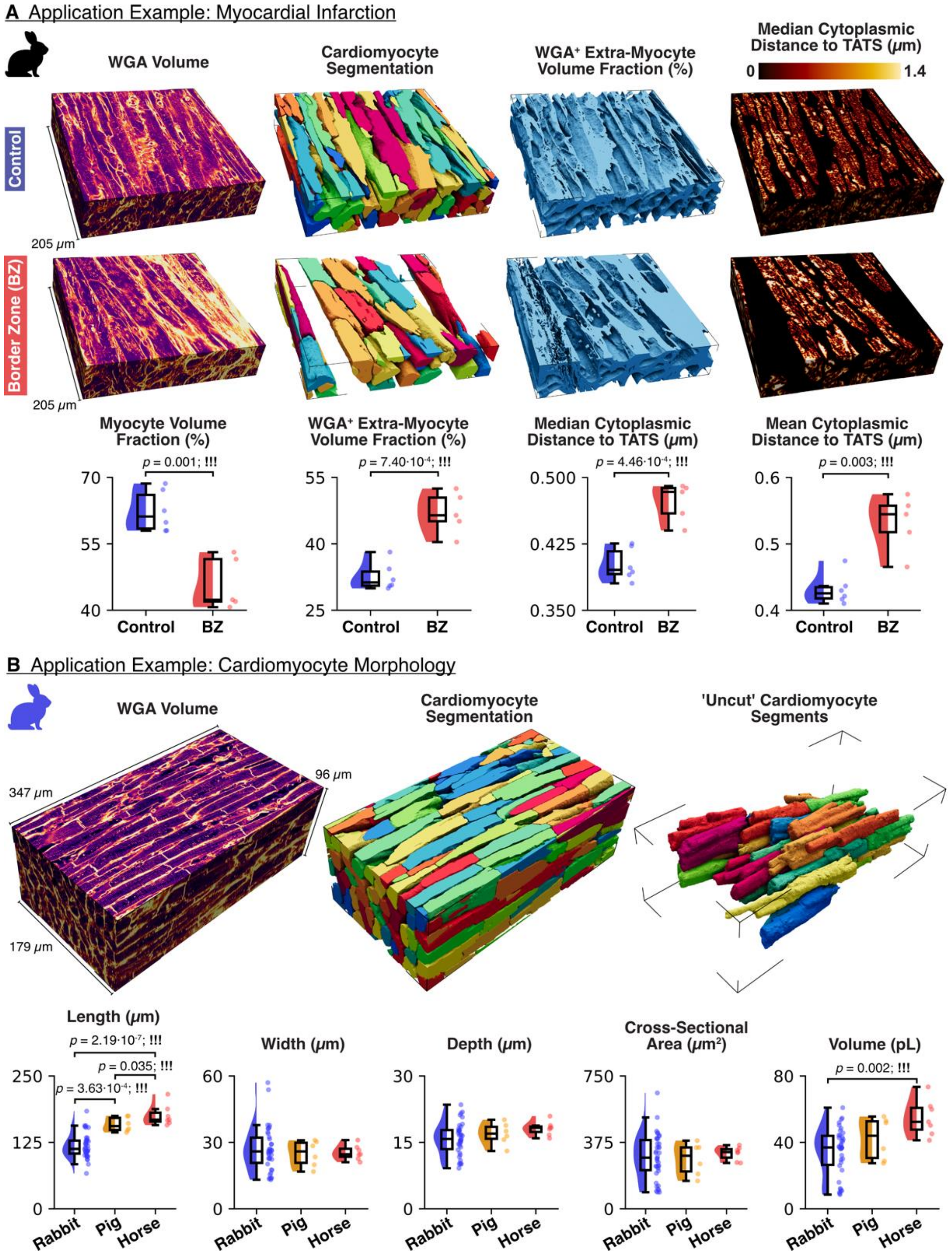

Fig. 2: Example analyses utilising cardiomyocyte segmentation. A: Analyses of microstructural tissue composition (myocyte and WGA-positive extra-myocyte volume fractions) and subcellular structures (mean/median distance of the cytoplasm to the nearest transverse-axial tubular system; TATS) within the context of cardiac (patho-)physiology. n = 6/5 image volumes for control and border zone, respectively. Image voxel size: 200 nm × 200 nm × 200 nm. **B:** Extensive morphological analyses of cardiomyocytes using proofread automated segmentations. Only cardiomyocytes that are not intersecting with the image volume boundaries were used for quantifications. n = 42, 7, and 6 cells for rabbit, horse, and pig, respectively. Image voxel size: 180–220 nm × 180–220 nm × 180 nm.

Reviewer 1:
What level of precision, both in time and space, does the new approach by the authors need to achieve? The take home message to non-imaging communities could be refined in my opinion.

Answer:
The toolkit, in its current form, segments static (fixed) tissue volumes; therefore, only spatial resolution is an essential consideration at this stage. For optimal and reliable performance of our toolkit, we firmly recommend near-isotropic voxel sizes of ~$(200\ \text{nm})^3$, matching the spatial sampling of our training data, which should be achievable when using most standard confocal microscopes.

If the framework were to be extended to live tissue studies, the required temporal precision would depend on the heart rate of the various species. For mice, we estimate that one would need to sample at >300 Hz (10 frames within a time-to-peak-contraction window of ~30 ms), for a whale, 20 Hz may be sufficient (10 frames within a time-to-peak-contraction window of >500 ms). These theoretical sampling rates would not need to be sustained throughout the contractile cycle, could be multiplexed, yet would be associated with a host of other challenges, such as motion-induced blur – aspects that are not part of the present study.

We modified the manuscript to state the needed spatial resolution clearly, with a take-home message for the non-imaging community:

Revision (Discussion, third paragraph):
For the acquisition of new image volumes, we recommend an isotropic voxel size of 200 nm to match our training data closely. Thus, a standard confocal or two-photon microscope should suffice; no specialised hardware is necessary.

Reviewer 1:
Do the authors think that their method can be applied to other high-resolution imaging modalities such as micro-CT or OCT?

Answer:
The workflows described here should be agnostic to the image modality used to generate data. Therefore, we believe the methods should generalise to other modalities if structural features (boundaries) can be identified with sufficient quality. Current micro-CT and OCT imaging do not have a resolution high enough to resolve the boundaries of individual cardiomyocytes, so a direct one-to-one implementation is likely to fail. However, for targets with larger anatomical features that have clear delineation, such as larger vessels or cardiac sheetlets, our presented strategies should work. In fact, we like to highlight that the implementation we utilise for the MultiCut agglomeration was originally built for large-scale electron microscopy [Beier17]. We have already applied the presented strategy to other image modalities, for example, to the segmentation of organelles in electron tomographic images (Fig. 3). PlantSeg, a pipeline conceptually similar to DTWS-MC, has also been applied very successfully to light sheet microscopy.

We have added this statement to the discussion (second to last paragraph):

Revision (Discussion, second to last paragraph):
Of note, we expect the segmentation workflows to generalise to different segmentation tasks and image modalities, as long as strong boundary features of biological structures are discernible at the imaging data resolution.

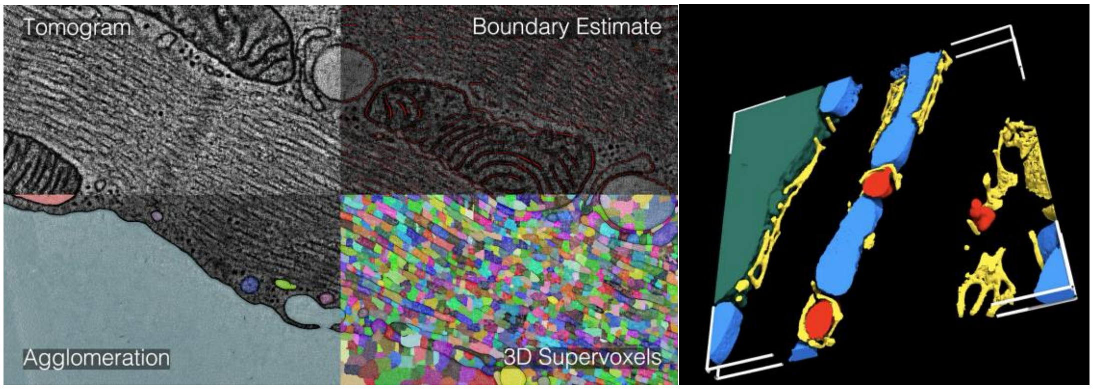

Fig. 3: Application of DTWS-MC and MultiCut to electron tomography image volumes to characterise the ultrastructure of cardiomyocytes. **Left:** Workflow steps: electron tomogram, boundary estimate, watershed-based 3D super voxels, and supervoxel agglomeration (feasible with MultiCut and semi-automatically with SegmentPuzzler). **Right:** 3D rendering of segmented membrane compartments: extracellular space (dark green), mitochondria (blue), sarcoplasmic reticulum (yellow) and transverse tubules (red). This figure contains unpublished data provided solely for the rebuttal letter and is not part of the revised manuscript.

References:

[Beier17] Beier T, Pape C, Rahaman N, Prange T, Berg S, Bock DD, Cardona A, Knott GW, Plaza SM, Scheffer LK, Koethe U. Multicut brings automated neurite segmentation closer to human performance. Nature Methods. 2017;14(2):101-2.

Reviewer 1:
Can the algorithm differentiate between multiple cell types in a volume of tissue based on shape? For example, Purkinje cells versus ventricular myocytes in the ventricular myocardium?

Answer:
We currently focus on solving the instance segmentation of working cardiomyocytes within the toolkit, and we have not performed any sub-classifications of cardiomyocytes yet. In the future, it is feasible to develop cell classification strategies as downstream applications of the toolkit.

Possible strategies could be:

1. Features based on 3D morphology (volume, surface-to-volume ratio, branching-related measures) can be extracted to train a machine learning classifier (e.g. a random forest or a support vector machine).
2. One could reformulate the classification problem as a 3D semantic segmentation problem, where a convolutional neural network predicts a binary Purkinje cell mask, analogous to how a cardiomyocyte mask is predicted in the DTWS-MC method. Notably, with this strategy, humans do not have to manually select features, as they will be automatically optimised in the neural network's training phase.

We would expect especially the latter strategy to be a powerful classifier, and have modified the discussion to disseminate the idea:

Revision (Discussion):
We identified DTWS-MC as the best-performing approach. **In our pipeline, a semantic cardiomyocyte mask is used to reduce merge errors with the extra-myocyte space. Future work may also introduce further semantic masks, e.g. to distinguish Purkinje or pacemaker cells, adding further granularity to the cardiomyocyte segmentation. DTWS-MC** works particularly well with SegmentPuzzler, as it has a higher split error, relative to its merge error, which can be easily corrected using our tool.

Reviewer 1:
Line 47: I had trouble finding the cells/minute results in the manuscript.

Answer:

The cells/minute figure was an aggregate from the throughput stated in the results text. We agree that this should be stated more clearly and have changed the manuscript accordingly:

Revision:
The resulting segmentations were proofread using SegmentPuzzler, requiring 28 and 76 minutes for the two stacks containing 82 and 232 cardiomyocytes, respectively**, resulting in a combined throughput of 3 cells/minute.**

Reviewer 1:
Line 79-92: Have the authors directly compared their new approach with the benchmarks of the previous approaches on lines 79-92 in the introduction.

Answer:
Context (lines 79–92):
Early work on 3D cardiomyocyte segmentation used manual deformation of surface meshes, followed by iterative thresholding (Lasher et al., 2009). Interactive deformation is time-consuming, with a total workload of 1–2 weeks per image volume (Seidel et al., 2013). Later studies replaced the mesh deformation with watershed-based partitioning and manual agglomeration, reducing the required effort to 5–10 hours for a similarly sized image volume (Seidel et al., 2013; Greiner et al., 2018). Parallel efforts achieved an estimated reconstruction throughput of 300–400 cardiomyocytes per workday in 40 µm-thick paraffin sections, using a commercially-available analysis software (Bensley et al., 2016). However, due to the processing steps for paraffin sections, capillary collapse and tissue shrinkage (e.g. during sample dehydration) may have accentuated cardiomyocyte boundaries. Additionally, the focus of prior work was on fully contained cardiomyocytes – rather than on reconstructing all cardiomyocytes in a given tissue volume – which may have eased the segmentation effort compared to whole-volume reconstructions of non-dehydrated, compression-free mounted tissue (Seidel et al., 2016).

Generally, establishing an open benchmark for automatic segmentation methods was a primary motivation for this project. In fact, we invited one of the authors, Fabio Frangiamore, and his independently developed algorithm (DTWS-MO), to join this project and contribute to the benchmarking effort presented in our results section. We believe that benchmarking ultimately leads to better-performing methods, and open benchmarks – both in terms of data and algorithms – are crucial. Unfortunately, [Lasher09], [Seidel13], and [Bensley16] are manual or semi-automatic methods that both heavily depend on users' experience, and they are immensely time-consuming, making a direct and fair comparison challenging. We therefore decided to benchmark our two independently developed segmentation algorithms against other automatic methods, namely Cellpose, Omnipose, and PlantSeg, as currently done in the manuscript. Notably, we have also benchmarked the time effort for the correction phase using SegmentPuzzler, but we omit comparisons to other tools, as these are again likely to be highly dependent on the operators' skills and familiarity with the software, and we would not be able to present other solutions fairly. Compared to previous approaches (hours to weeks per volume), a proofreading speed of ~3 cells per minute sounds beneficial to us.

Reviewer 1:
Line 100: "SegmentPuzzler", is this separate from the automatic segmentations mentioned earlier in the abstract? I imagine the methods will explain this, but to this point it is unclear to me the difference/reason for doing both automated and semi-automated segementation techniques. Is SegmentPuzzler basically a user-friendly interface to perform and/or visualize the segmentation of cardiomyocytes?

Answer:
Yes, exactly. SegmentPuzzler is primarily designed to correct automatically generated segmentations, but it also has additional uses for post-processing and refining segmentations, such as morphological processing and segment extraction based on volume, overlap, or annotation. We have revised the manuscript to make these points clearer.

Revision (Introduction):
To address these challenges, we developed and validated a comprehensive, deep learning-enabled toolkit for 3D instance reconstruction of cardiomyocytes in confocal microscopy volumes, **and**

additionally introduce an interactive segmentation tool with a graphical user interface called 'SegmentPuzzler'. This tool facilitates fast annotation and proofreading of segmentations based on clustered volumetric pixels known as supervoxels.

Reviewer 1:
Line 103: Is the major flaw of the previous approaches listed in the introduction the input data (quality, resolution, preparation, etc.) or from shortcomings in the algorithms used in their methods? For example, would past methods also work better with higher quality data, as the authors appear to do with their image restoration method? I imagine there is also a balance between processing time and accuracy.

Answer:
While this is difficult to answer definitively, we believe that both the application of deep learning in the segmentation arm of our work, as well as the improved sample preparation and image quality, contributed significantly to the success of our framework.

We agree that there is a balance between processing time and accuracy. However, we do not believe that the processing time of DTWS-MC is currently prohibitive; in fact, we would not hesitate to increase the processing time (from minutes to multiple hours or days) if that would substantially improve the algorithm's performance. We benchmarked the wall-clock time of the three best-performing algorithms, DTWS-MC, Omnipose, and PlantSeg, in Table 1 (new manuscript Appendix Table A2). The current end-to-end runtime of the algorithm is 61 minutes for a relatively large stack of 2,778 × 1,016 × 280 voxels; this can be lowered to 17 minutes by disabling test-time augmentation (DTWS-MC-no-TTA). Please note that for Omnipose, we downsampled the image with a factor of 3 in each dimension to increase the receptive field of the model, as recommended in Omnipose's documentation. This, however, reduces the number of voxels in the volume by a factor of 27, which is the most likely reason for its fast runtime.

TABLE 1
SEGMENTATION RUN TIME (~800 MILLION VOXEL VOLUME)

Method	Time (min)	Adapted Rand Error
Omnipose	**3.92**	0.11
PlantSeg	14.60	0.10
DTWS-MC	60.82	**0.06**
DTWS-MC-no-TTA	16.73	0.07

Best observed metric is marked in bold. TTA: test-time augmentation. Testing was conducted on a computer equipped with an Intel i9-13900K processor, 128GB of RAM, and an Nvidia RTX 4090 graphics card. We report the average from three runs. Timing was recorded on an image stack with 2,778 × 1,016 × 280 voxels, representing a larger-than-average image stack.

Reviewer 1:
Line 119: Is each data set number I,II, and III linked to the data in Table 1?

Answer:
Yes, even though Datasets may have multiple entries in Table 1. We added clarifications to the text to resolve ambiguities.

Reviewer 1:
Lines 169, 174, 189: There are different out-of-plane and voxel resolutions between the different datasets. What is the minimal resolution needed before the shape of the cells becomes distorted? This should likely be determined for the smallest cell across the 7 species.

Answer:
Similar to our explanation regarding the required spatial resolution, we recommend matching our training data with a near-isotropic voxel size of ~200 nm. Deviating from the resolutions by a larger factor may necessitate retraining of associated neural networks. If subcellular locations and details are not important, the resolution can be lowered to at least 600 nm (as for Omnipose, the input data is downsampled by a

factor of 3), and the overall shape of cardiomyocytes can still be resolved. We would like to refrain from speculating about the minimum resolution needed, as this, even for the species tested here, depends on the subject's age, the exact tissue sampling location, and the specific research question at hand. For example, we are also currently working on paediatric samples with congenital heart diseases, where a 200 nm resolution is strictly necessary to resolve ambiguities, as cardiomyocytes in these samples are smaller than those seen in adult samples.

Reviewer 1:
Line 219: What are the units of 101? Also, why 101?
Line 222: Again, what are the units of 150?

Answer:
Thank you for pointing this out. While window lengths are technically unitless, we revised the manuscript to improve the readability of this section. Generally, the smoothing and maximum filter is a very coarse signal equalisation technique across depth (z-axis), which was determined empirically. The first filter smooths the signal, and therefore, its length should be large enough that isolated bright structures (e.g. capillaries) do not lead to sharp peaks in the intensity profile. The second maximum filter bridges areas that are mostly void of tissue, for example, at the beginning and the end of the tissue section. Its values should be chosen to be larger than the maximum amount of mostly empty slices expected in the dataset:

Revision:
These resulting estimates were then smoothed along the z-axis using a Savitzky-Golay filter with a window length of 101 **(unitless index)**.

Revision:
The 1D threshold profile was then further filtered with a maximum filter **with a length of 150 (unitless index)** across the z-axis to ensure that a valid threshold was derived, even if the complete image plane contained only background noise.

Reviewer 1:
Line 331: For the various datasets, is it known how many cardiac myocytes are present on average in each species for 1um^3 of tissue?

Answer:
The cardiac myocyte density strongly depends on two parameters: ‘myocyte packing’, i.e., the fraction of tissue volume occupied by myocytes, and the individual myocyte size. Myocyte packing depends on sampled regions and potential presence of any remodelling within the heart, and we acquired image stacks that are not all densely filled with myocardium (e.g. due to tissue slices being placed not perfectly flat on a cover glass). However, we have calculated average values of cardiomyocyte length, width, depth, cross-sectional area, and volume in Table 2 (also new manuscript Table 4). That said, we would like to exercise caution in interpreting the results as a definitive characterisation of species-related properties. While we believe we can use our toolkit to assess 3D cardiomyocyte morphology in future work, a focused effort to create a suitable dataset is necessary, particularly one that matches age, sex, and sampling location across different species, and that uses a uniform protocol for tissue sampling and processing.

TABLE 2

MORPHOLOGICAL PROPERTIES OF CARDIOMYOCYTES (MEAN ± STANDARD DEVIATION)

Species	Study/Dataset	n	Length (µm)	Width (µm)	Depth (µm)	Volume (pL)	CSA (μm^2)
**Mouse**	*Struckman*	150	127.3$_a$	31.4$_a$	11.4$_a$	19.4$_a$	
	Dataset II$_2$	90	107.1 ± 25.4	16.1 ± 2.8	10.8 ± 1.7	11.9 ± 40.0	114.2 ± 28.2
**Rat**	*Satoh*	21	141.9 ± 14.9	32.0 ± 4.8	13.3 ± 1.6	34.4 ± 7.0	
	Natali: EPI S	240	121.3 ± 1.0	20.1± 0.2	13.9 ± 0.1	18.6 ± 0.4	
	Chen: Apex		137.0 ± 6.7	30.6 ± 2.1	12.0 ± 1.5	39,0 ± 3,5	286 ± 32
	Dataset II$_2$	150	124.3 ± 26.3	26.9 ± 5.8	16.3 ± 3.1	33.7 ± 11.0	279.0 ± 88.1
**Rabbit**	*Satoh*	14	123.8 ± 14.4	33.6 ± 6.7	12.8 ± 1.4	30.9 ± 5.2	
	Dataset II$_1$	38	118.1 ± 23.3	26.7 ± 10.1	15.8 ± 3.6	34.3 ± 14.5	292.2 ± 128.7
	Dataset II$_2$	60	120.7 ± 17.2	27.6 ± 9.3	15.3 ± 3.5	33.8 ± 14.9	284.5 ± 130.2
**Pig**	*Scott*		156.5$_a$	24.5$_a$	17.5$_a$		
	Dataset II$_1$	6	158.7 ± 13.6	25.0 ± 5.8	16.9 ± 2.5	42.1 ± 12.8	280.7 ± 92.1
	Dataset II$_2$	210	168.7 ± 31.5	31.6 ± 6.9	18.1 ± 3.1	54.7 ± 12.9	379.2 ± 98.5
**Horse**	Dataset II$_1$	7	175.9 ± 20.1	25.4 ± 3.3	18.1 ± 1.6	54.9 ± 11.0	312.8 ± 38.7
	Dataset II$_2$	180	162.7 ± 45.8	28.8 ± 6.3	17.6 ± 3.8	50.1 ± 17.7	321.9 ± 104.6
**Elephant**	Dataset II$_2$	22	111.5 ± 52.2	24.1 ± 10.6	13.6 ± 5.0	29.0 ± 26.2	215.5 ± 138.4

CSA: cross-sectional area; $_1$: only cells that do not intersect with the image volume boundaries were analysed; $_2$: top 30 cells by volume analysed per stack; $_a$: median value.

Reviewer 1:
Line 349: In addition to accuracy, I imagine that the time needed to perform the segmentation is also important. Can the authors comment on the speed of each approached tried with respect to accuracy?

Answer:
We agree that there is a trade-off between speed and accuracy (Table 1). However, at the current state, where few high-accuracy automatic segmentation methods for cardiomyocytes exist, processing speed/throughput is currently not a limiting factor. As stated before, we would not hesitate to increase the processing time if that would substantially reduce manual proofreading effort.

Reviewer 1:
Line 381: The main point of the results in this section are unclear to me.

Answer:
Context (line 381–389):
3.4 Proofreading Effort and Generalisation to Unseen Conditions

To assess the utility of our method for generalising to previously unseen species, we applied the best-performing DTWS-MC method to two stacks of human myocardium.

Thank you for pointing this out. We have added clarifications to the manuscript.

Revision:
Given the large variety of model organisms and experimental conditions under which cardiac research is conducted, we assessed to what extent our method, trained on datasets I and II, can be

generalised to a new set of data, dataset III, and quantify the effort needed to proofread the segmentation using SegmentPuzzler. Dataset III contains images of a species not trained on (human), and sample preparation and imaging were performed by different researchers than for datasets I and II. To quantify proofreading effort and assess generalisation, we focused on the best-performing DTWS-MC method. Notably, cell morphology differed considerably from the other datasets, and one of the volumes showed an unusually abrupt 90-degree change of cardiomyocyte orientation in the centre of the volume **(Appendix Fig. A2).**

Reviewer 1:
Line 404: The authors mention that their method and/or datasets could improve computational models. How so specifically?

Answer:
Many computational models require meshes of cardiac microstructure, and to the best of our knowledge, there are no publicly available datasets for this purpose. This is especially true when studying tissue electrics and mechanics, which often use synthetic geometries. For example, a commonly used approximation is square bricks or cylinders (of note: also widespread in cardiomyocyte illustrations). By contrast, our images show cardiomyocytes with a width-to-height aspect ratio of approximately 1:2–1:3 (also earlier reports, see Table 2, [Nishimura04]). We expect that having both the method to generate realistic and high-resolution meshes, as well as datasets of already segmented myocardium, openly available, will facilitate simulation with realistic microstructural geometries.

As for concrete examples, we have published *in silico* methods that utilise such image data/reconstructions to inform computational modellers and cardiac researchers [Greiner18, Greiner22]. We also believe that microstructural EMI models show fantastic potential, especially when combined with realistic geometries [Jæger23]. Our data is currently also used in the EuroHPC-funded center of excellence MICROCARD, which works on simulating cardiac electrophysiology on the sub-cellular scale on exascale systems. Finally, using the datasets described in our paper, we have established collaborations with computational modellers to conduct *in silico* experiments on microstructural myocardial mechanics.

References:
[Greiner22] Greiner J, Sankarankutty AC, Seidel T, Sachse FB. Confocal microscopy-based estimation of intracellular conductivities in myocardium for modeling of the normal and infarcted heart. Computers in Biology and Medicine. 2022;146:105579.
[Greiner18] Greiner J, Sankarankutty AC, Seemann G, Seidel T, Sachse FB. Confocal microscopy-based estimation of parameters for computational modeling of electrical conduction in the normal and infarcted heart. Frontiers in Physiology. 2018;9:239
[Nishimura04] Nishimura S, Yasuda SI, Katoh M, Yamada KP, Yamashita H, Saeki Y, Sunagawa K, Nagai R, Hisada T, Sugiura S. Single cell mechanics of rat cardiomyocytes under isometric, unloaded, and physiologically loaded conditions. American Journal of Physiology-Heart and Circulatory Physiology. 2004;287(1):H196-202.
[Jæger23] Jæger KH, Tveito A. Efficient, cell-based simulations of cardiac electrophysiology; The Kirchhoff Network Model (KNM). NPJ Systems Biology and Applications. 2023;9(1):25.

Reviewer 1:
Line 457: I think some of these time benchmarks could be moved to the results section.

Answer:
We have revised the text to clarify that these values were already reported in the results section, and are restated in the discussion to provide context.

Reviewer 1:
Line 464: Could SegmentPuzzler improve the diagnosis/treatment of cardiac abnormalities associated with cellular hypertrophy? If so, what specific type of information would be transferred to a clinician or their technicians?

Answer:
The primary goal of our toolkit is to strengthen basic cardiac science, which may ultimately improve the diagnosis/treatment of cardiac diseases. Nevertheless, aiding clinical decision-making directly holds promise, as manual 2D histopathological assessment already provides key information such as myocyte disarray, hypertrophy, and degree of interstitial fibrosis, to clinicians today [Alashi19]. Using a fully automated 3D analysis should be advantageous, particularly due to the reduced presence of sectioning artefacts (see Fig. 1), improved intra-individual sampling, and minimised inter-observer differences and manual labour. In a study of collagen accumulation in myectomies from patients with tetralogy of Fallot, we have previously observed large intra-individual heterogeneities and demonstrated that sampling of an individual 2D tissue slice is insufficient for reliable analyses [Wülfers21]. We expect similar conclusions for the assessment of cellular hypertrophy.

References:
[Alashi19] Alashi A, Desai RM, Khullar T, Hodges K, Rodriguez ER, Tan C, Popovic ZB, Thamilarasan M, Wierup P, Lever HM, Smedira NG. Different histopathologic diagnoses in patients with clinically diagnosed hypertrophic cardiomyopathy after surgical myectomy. Circulation. 2019;140(4):344-6.
[Wülfers21] Wülfers EM, Greiner J, Giese M, Madl J, Kroll J, Stiller B, Kohl P, Rog-Zielinska EA, Fürniss HE. Quantitative collagen assessment in right ventricular myectomies from patients with tetralogy of Fallot. EP Europace. 2021;23(Supplement_1):i38-47.

Point-by-point response to Reviewer 2:

Reviewer 2:
In this manuscript the authors present a freely available toolkit for the automatic segmentation of 3-D confocal Z stacks from cardiomyocytes. I agreed with the authors that a 3-D view of the myocardium is extremely useful for understanding cardiac pathophysiology and that analytical toolkits are currently insufficient. Researchers often rely on manual segmentation which is not time or cost-effective. Therefore, new ways to understand the 3-D architecture of cardiac tissue would be welcomed in the field. I would have thought therefore that this study would be interesting for those working on the structure of the myocardium.

The methods implemented in the design of the toolkit are not within my area of expertise but the authors do seem to have taken great care with the development. For example, 2 types of 3-D segmentation strategies were used firstly looking at XY slices and secondly using YZ and XZ slices. It's also interesting to see the use of different species, disease states and the inclusion of cultured myocardium for the evaluation.

Answer:
We would like to thank the reviewer for their comprehensive and constructive feedback, as well as for the overall positive evaluation of the manuscript.

Reviewer 2:
The manuscript is by necessity technical. However, given its target audience, the authors may wish to increase accessibility generally to help the end user understand how the toolkit works and where it strengths and weaknesses lie. With this in mind it would be helpful to have brief explanations of key terms such as supervoxel, Rand error, split error, merge error and watershed algorithm for the non-specialist reader.

Answer:
Thank you very much for pointing that out. We have tried to make the manuscript more accessible by revising it and adding a new glossary of key image segmentation-related terminology explained in plain language (new Appendix Table A1):

Revision (footnote in the first paragraph):
Of note, for readers unfamiliar with image segmentation terminology, we provide a plain-language glossary of key terms in Appendix Table A1.

Additionally, we hope that the provided web visualisations will aid non-technical readers in assessing our toolkit's performance in an intuitive and unbiased manner, and provide stronger callouts to them.

Reviewer 2:
In figure 2, for the cells in the annotations, it would be useful to relate them to the XY image shown above. Maybe this could be achieved with coloured asterisks in the XY image to highlight particular cells to the reader?

Answer:
We agree that this would be a nice addition to the figure. We have experimented with different annotation strategies, but as the WGA signal is only shown in a central rectangular crop area, and the 3D rendering primarily shows the outermost cells, finding prominent cells in both views is challenging. However, we found the interactive Supplementary Web Visualisation S1 (Fig. 4), especially with the function to pause at a specific z-index, to be a powerful tool for relating image data and overall segmentation. We have revised the image legend to actively guide the reader towards it.

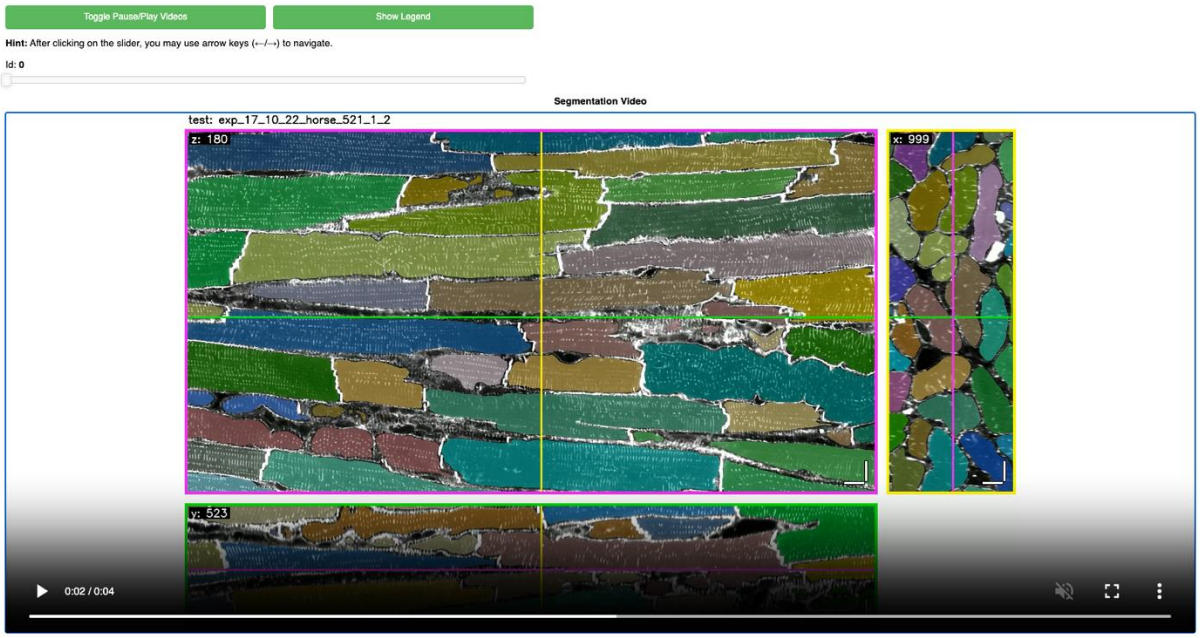

Fig. 4: Frames from the Supplementary Web Visualisation S1. The top-left green button can be used to pause the visualisation. The three orthogonal views can be used to explore and relate the annotations to the original image. Scalebar: 10 µm.

Revision:
Fig. 2. 3D segmentation and xy/xz views of WGA-labelled myocardium across diverse experimental conditions and species, including control myocardium, myocardium post-myocardial infarction, and myocardium after *ex vivo* tissue culture for 7 days. For large volumes, cropped images are shown in XY. All image stacks with their corresponding annotation used in our study are visualised in Supplementary Web Visualisation S1. **We recommend inspecting the web visualisations, complementing the static version above, for exploration of image and annotation data. The visualisation can be viewed in a browser without special software.**

Reviewer 2:
In figure 3 the legend is very helpful and takes the reader through the process the authors have employed. However, in the results text this is less clear. Could the authors add more detail to the results text to guide the reader through the data in more detail making reference to the separate panels in figure 3? It would also help in table 3 to make it clear that the merge and split columns refer to errors.

Answer:
Thank you for the suggestion. We have revised the text to include callouts to individual figure panels and have updated Table 3 to explicitly state Merge and Split errors.

Reviewer 2:
For figure 4 it would also be useful to mention the specific panels in figure for in the results section to help guide the non-specialist reader.

Answer:
We agree and have revised the manuscript accordingly.

Reviewer 2:
At line 365 the authors state DTWS - MC and PlantSeg produced obvious split errors especially in regions containing branching cardio myocytes. It is not clear to me exactly what the authors are referring to in panel B. Could the author show these errors? Perhaps by zooming in and explaining in the results text? Would this be helpful to potential users so they could spot errors in their own data sets? It would also be useful to see examples of cells which falsely extend into the extra cellular space. Similarly, it would be helpful for the authors to highlight to the reader examples of merge errors.

Answer:
Thank you for pointing out that these terms were not explained well enough. We have revised error descriptions in the manuscript and show clearer examples of the different error types with zoom-ins in Fig. 5 (new manuscript Appendix Fig. A1).

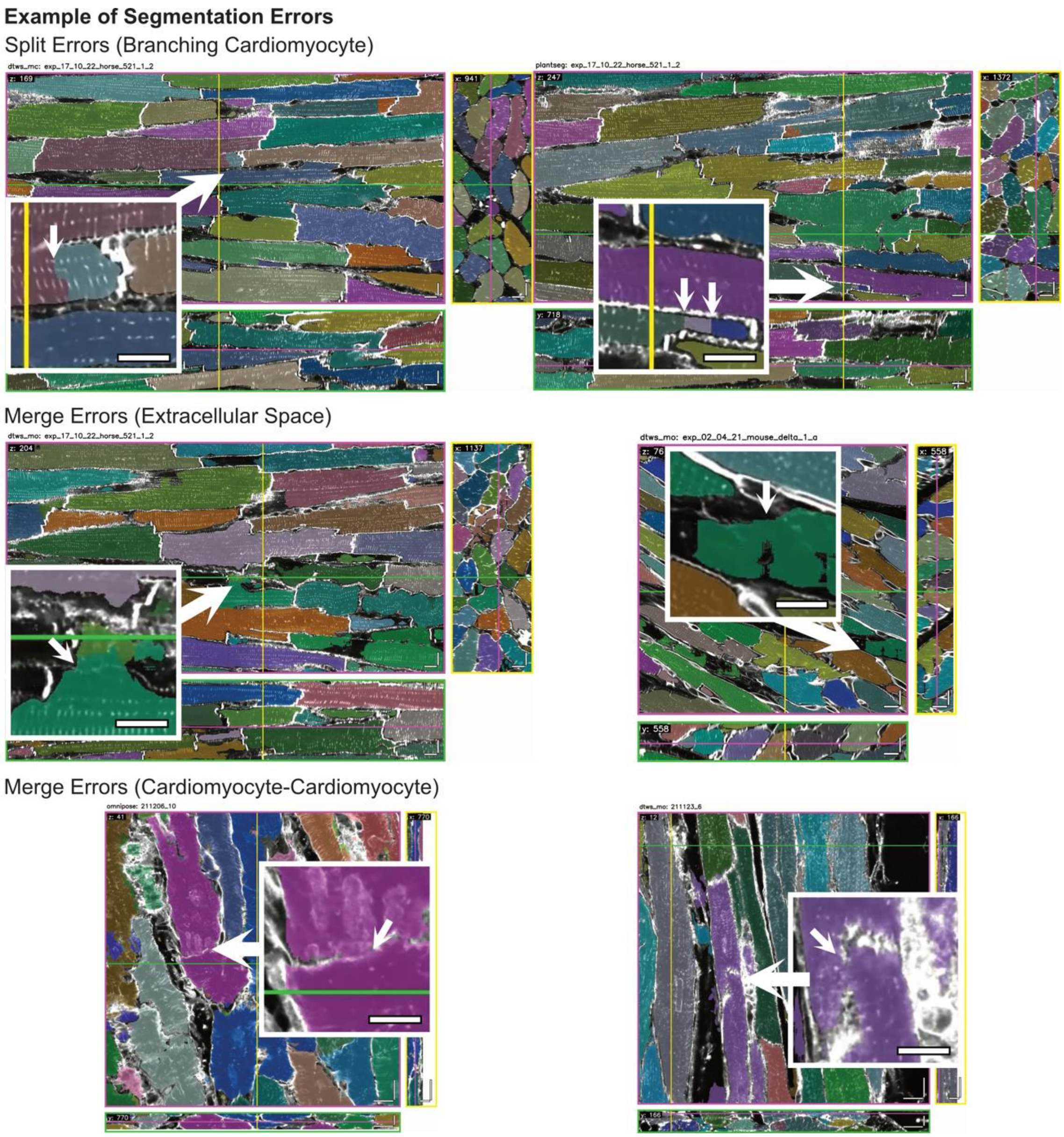

Fig. 5: Examples of split and merge errors in the context of cardiomyocyte segmentation, taken from the Supplementary Web Visualisation S3. Arrows point towards the error location, with one error per image shown as a magnified insert. Scalebar: 10 µm.

Reviewer 2:
In section 3.4 the authors have assessed the utility of their method by applying the best performing method to 2 stacks of human myocardium. While the authors discuss this and give their calculated errors, they should also present the data in the form of a figure. It would be useful to discuss what the errors using the best segmentation method means for the data output in a little more detail.

Answer:
We have created a new figure highlighting the data and the unique morphology (Fig. 6, new manuscript Appendix Fig. A2). Additionally, we have added details to the discussion regarding the achieved accuracy of the segmentation algorithms. To demonstrate the practical implications of these errors, we quantified

errors in downstream applications, which are explained in greater detail in the next two answers to the reviewer.

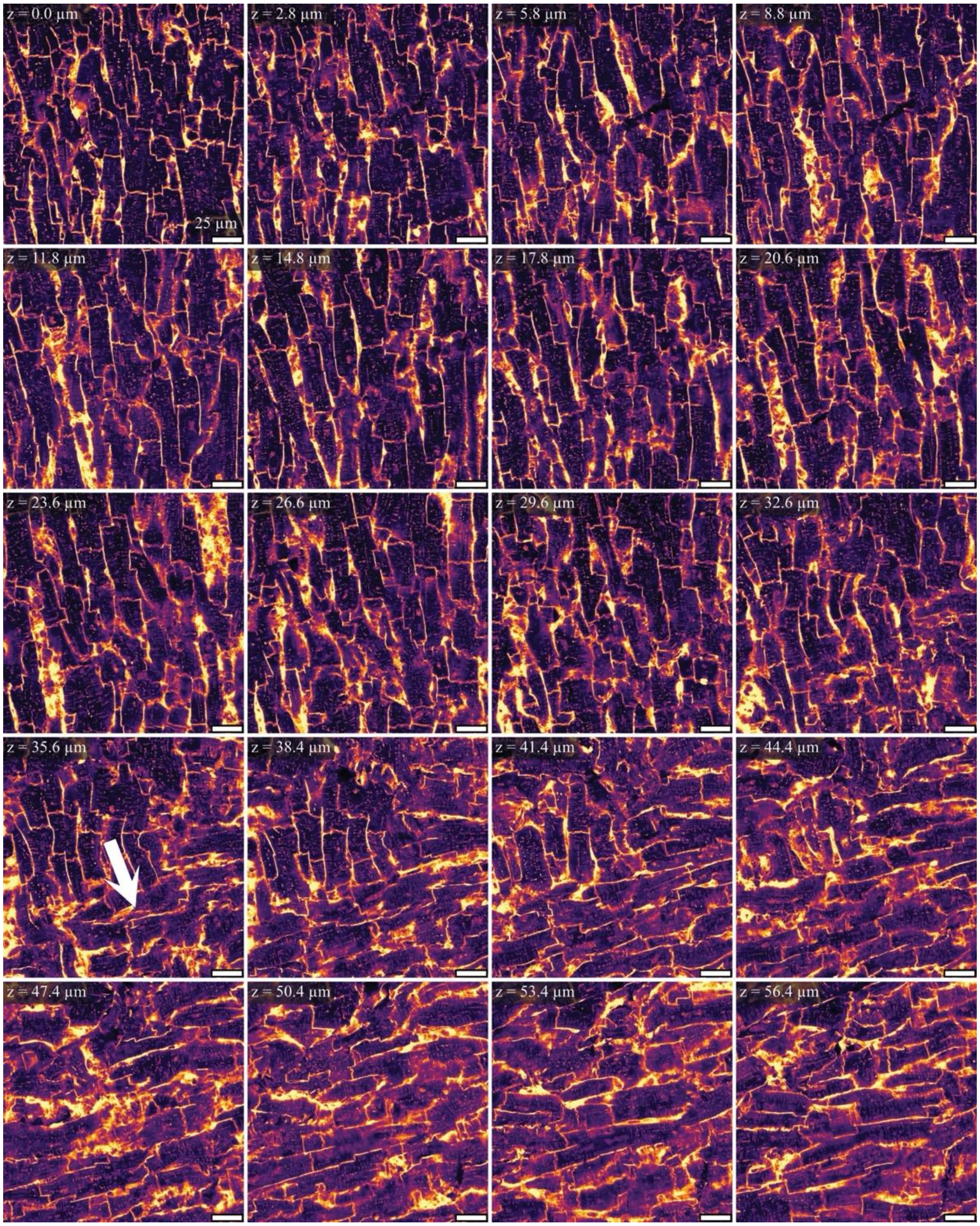

Fig. 6: Exemplary test data from healthy human ventricular myocardium (rejected donor hearts). The donors were individuals who had passed away from causes unrelated to cardiac diseases. A ~90° cardiomyocyte orientation change starts around 35.6 µm (white arrow). Such cardiomyocyte orientation changes are not present in the training dataset. Scale bar: 25 µm.

Reviewer 2:
The data in figure 3D is striking and it appears that the tool kit would be very useful for those interested in understanding subcellular structures in 3-Dimensions and in the case of the images presented, the transverse tubule network. Please could the authors include more information about whether this is possible with their toolkit? For example could the toolkit assess transverse tubule density within a cell or are there problems with thresholding? Could the end user expand the utility of the tool kit for data sets of other cellular components e.g. mitochondria?

Reviewer 2 (part of next comment):
(...) If the toolkit can be used for sub-cellular analysis this could include transverse tubule density or how transverse tubule density changes with disease (this could be compared to published data).

Answer:
We thank the reviewer for the very positive assessment of the data in Figure 3D. Indeed, we believe that cardiomyocyte segmentation is highly useful for addressing a range of research questions. Subcellular structures and 3D analyses are a clear downstream application that our framework helps to enable. To demonstrate this, we have implemented an analysis of TATS density in rabbit control myocardium and post-infarction tissue (Fig. 2; new manuscript Fig. 6). Thresholding is not an issue. Extension to other (sub-)cellular components, such as mitochondria, is feasible, provided that the components are fluorescently marked with sufficient signal quality. We have extensively revised the manuscript, including new sections for Methods (Section 2.8) and Results (Section 3.5). We have not found published data on TATS density for ventricular rabbit myocardium after myocardial infarction, and have therefore omitted the comparison. We do, however, include literature comparisons on the morphological properties of cardiomyocytes, as shown in later reviewer responses.

Reviewer 2:
To further reassure future users of the software, are the authors able to make measurements from their data sets and compare it to measurements using a manual method? This could for example be a count of the number of cells in a given volume, measurements of cell length or width of volume et cetera. If the toolkit can be used for sub-cellular analysis this could include transverse tubule density or how transverse tubule density changes with disease (this could be compared to published data).

Answer:
We agree with the reviewers’ suggestion to reassure future users of the software. Manual methods to validate length, width, or volumes are, however, challenging. One main pitfall is the lack of precise control over the cutting plane, as demonstrated in Fig. 1, above (new manuscript Fig. 1). We also note that in real cardiomyocytes, the geometry is significantly more complex than that of a ’brick’, further complicating the mathematically exact measurement of 3D length, width, and height.

Thus, 3D measurement quickly becomes time-intensive and more of a measure of the annotator’s skill than a verification of the software. We therefore prefer to perform these measurements with the assistance of a 3D segmentation mask, as this now allows for mathematically precise measurements compared to ‘fully manual’ assessments. To verify that the analysis process was correctly implemented, we generated a block of known length, width, and height, and checked the generated errors for the calculated length, width, height, cross-sectional area, and volume for different block orientations. Errors were negligible with values in the range of 10^{-10} µm or less, presumably arising from the floating-point number representation. Assuming that one now trusts the analysis of these masks, proofreading of the segmentation itself may reassure readers that the extracted morphological data is trustworthy. For the data presented in our paper, this can be easily done visually using the Supplementary Web Visualisation S1 by checking whether segmentation outlines neatly match the overlaid WGA signal. Analogously, manually counting the number of cells in a given volume should be equivalent to proofreading the 3D segmentation. We excluded very small segments (smaller than 3 pL) that arise from intersection with the imaging border, as here it is often unclear whether neighbouring fragments belong to the same cardiomyocyte or not.

With these prerequisites, we developed the following strategy to reassure future users:

1. Table 2 (above; new manuscript Table 4) compares cardiomyocyte morphology, including length, width, height, cross-sectional area, and volume.
2. Figure 7 (new manuscript Appendix Fig. A4) and Table 3 (new manuscript Appendix Table A3) illustrate the impact of errors of automatic segmentation methods on the aforementioned morphological features or cardiomyocyte count.

TABLE 3

MORPHOLOGICAL PROPERTIES OF CARDIOMYOCYTES (TEST SET)

Method	Cell Count (Cells)	Length (μm)	Width (μm)	Height (μm)	Volume (pL)	CSA (μm^2)
Manual Proofreading	70.07 ± 50.60	109.89 ± 35.51	22.27 ± 6.36	11.22 ± 5.35	23.69 ± 18.20	181.22 ± 124.72
Omnipose	68.71 ± 51.17	110.66 ± 35.96	23.09 ± 6.72	12.33 ± 5.45	26.82 ± 20.61	199.09 ± 136.30
DTWS-MO	64.36 ± 47.09	114.42 ± 39.34	24.22 ± 7.60	13.06 ± 5.85	28.73 ± 22.55	202.68 ± 142.02
PlantSeg	69.93 ± 50.33	113.87 ± 45.57	22.24 ± 6.32	11.26 ± 5.30	23.48 ± 18.43	182.14 ± 123.91
DTWS-MC	72.79 ± 53.19	107.99 ± 36.49	22.24 ± 6.26	11.19 ± 5.25	23.40 ± 18.22	182.09 ± 123.58

CSA: cross-sectional area. n = 14 image volumes.

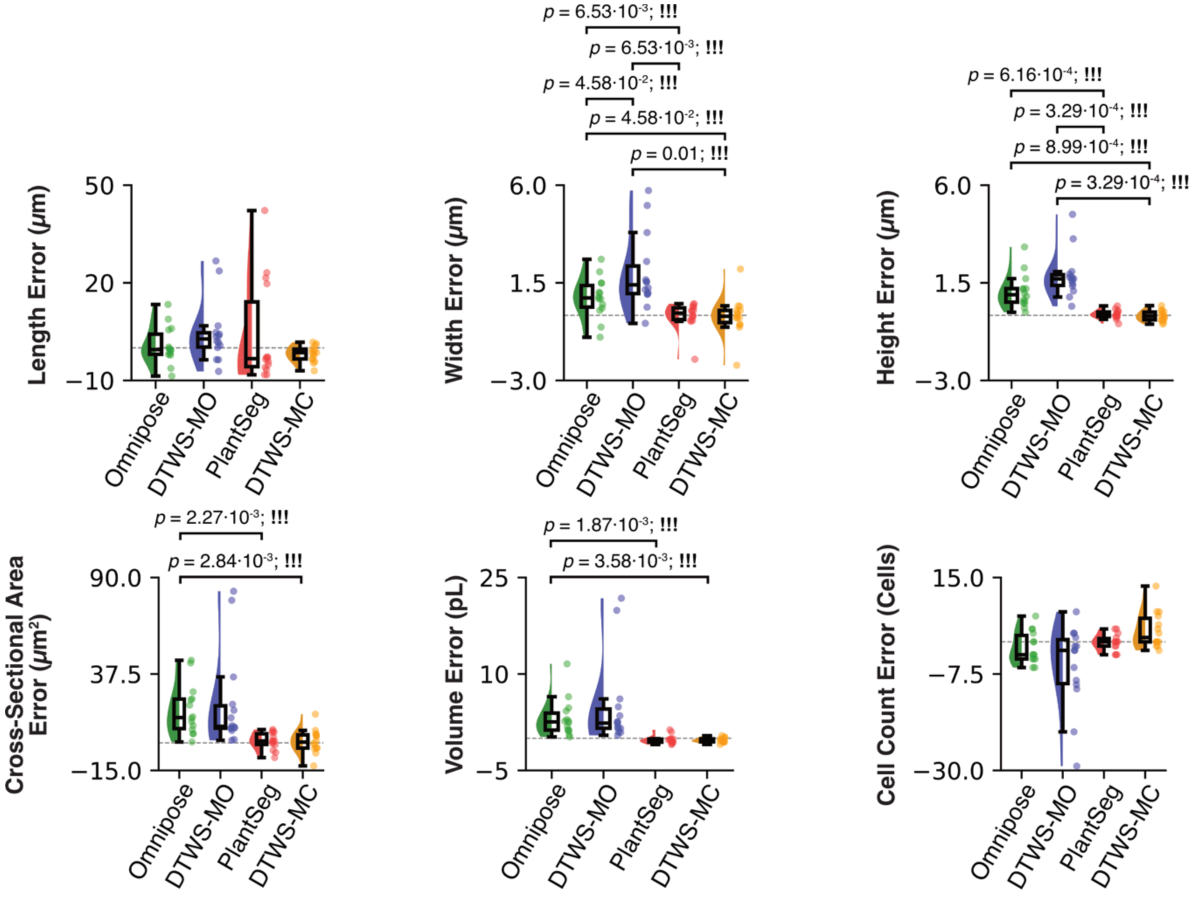

Fig. 7: Errors of automated cardiomyocyte segmentation strategies in a morphological analysis (n = 14 image volumes). The dashed lines represent an error of 0. Small, medium, and large effect sizes are indicated by "!", "!!", and "!!!", respectively.

Reviewer 2:
Did the authors have any problems with WGA staining the golgi in their datasets and if so how was this accounted for?

Answer:
We frequently observed WGA staining in perinuclear regions (Fig. 8, and Supplementary Web Visualisation S1), consistent with the Golgi apparatus [Fishbein24]; however, without additional staining, we can't confirm their identity with certainty. To date, this property of WGA has not caused issues for the segmentation of cardiomyocytes, and therefore, we have not accounted for it. In cases where this could be problematic, we suggest that an additional DNA stain, e.g. DAPI, and morphological operations (repeated dilation into WGA-positive space, starting from the segmented nucleus) could be used to segment potentially problematic areas.

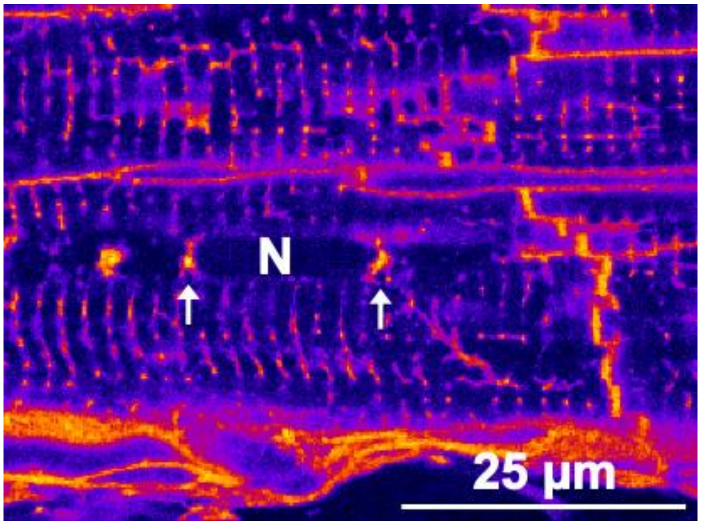

Fig. 8: Perinuclear WGA-positive structure, possibly related to the Golgi apparatus, in control left-ventricular rabbit tissue slices. N: nucleus, arrows point to a structure which could be the Golgi apparatus.

We refined the manuscript to mention staining of the Golgi apparatus:

Revision (Discussion – WGA limitations):
An additional limitation comes from the fact that WGA does not bind to the cardiomyocyte membranes directly; instead, it binds to components of the glycocalyx on the cell surface. **Of note, we frequently observed WGA staining in perinuclear regions, consistent with the location of the Golgi apparatus; however, without additional staining, we can't confirm the identity of these structures with certainty.**

References:
[Fishbein24] Fishbein GA, Bois MC, d'Amati G, Glass C, Masuelli L, Rodriguez ER, Seidman MA. Ultrastructural Cardiac Pathology: The Wide (yet so very small) World of Cardiac Electron Microscopy. Cardiovascular Pathology. 2024:107670.

Dear Dr Greiner,

Re: JP-RP-2025-288557R1 "A Deep Learning-Enabled Toolkit for the 3D Segmentation of Ventricular Cardiomyocytes" by Joachim Greiner, Fabio Frangiamore, Frédéric Sonak, Josef Madl, Thomas Seidel, Peter Kohl, and Eva A. Rog-Zielinska

Thank you for submitting your manuscript to The Journal of Physiology. It has been assessed by a Reviewing Editor and by 3 expert referees and we are pleased to tell you that it is acceptable for publication following satisfactory revision.

The referee reports are copied at the end of this email.

Please address all the points raised and incorporate all requested revisions or explain in your Response to Referees why a change has not been made. We hope you will find the comments helpful and that you will be able to return your revised manuscript within 4 weeks. If you require longer than this, please contact journal staff: jp@physoc.org.

Your revised manuscript should be submitted online using the link in your Author Tasks Link Not Available. This link is accessible via your account as Corresponding Author; it is not available to your co-authors. If this presents a problem, please contact journal staff (jp@physoc.org). Image files from the previous version are retained on the system. Please ensure you replace or remove any files that are being revised.

If you do not wish to submit a revised version of your manuscript, you must inform our journal staff (jp@physoc.org) or reply to this email to request withdrawal. Please note that a manuscript must be formally withdrawn from the peer review process at one journal before it may be submitted to another journal.

TRANSPARENT PEER REVIEW POLICY: To improve the transparency of its peer review process The Journal of Physiology publishes online, as supporting information, the peer review history of all articles accepted for publication. Readers will have access to decision letters, including Editors' comments and referee reports, for each version of the manuscript, as well as any author responses to peer review comments. Referees can decide whether or not they wish to be named on the peer review history document.

ABSTRACT FIGURES: Authors are expected to use The Journal's premium BioRender account to create/redraw their Abstract Figures. Information on how to access this account is here: https://physoc.onlinelibrary.wiley.com/journal/14697793/biorender-access.

This will enable Authors to create and download high-resolution figures. If authors have used the free BioRender service, they can use the instructions provided in the link above to download a high-resolution version suitable for publication.

The link provided should only be used for the purposes of this submission. Authors will be charged for figures created on this account if they are not related to this manuscript submission.

LANGUAGE EDITING AND SUPPORT FOR PUBLICATION: If you would like help with English language editing, or other article preparation support, Wiley Editing Services offers expert help, including English Language Editing, as well as translation, manuscript formatting, and figure formatting at www.wileyauthors.com/eeo/preparation. You can also find resources for Preparing Your Article for general guidance about writing and preparing your manuscript at www.wileyauthors.com/eeo/prepresources.

RIGOUR AND REPRODUCIBILITY: We recommend authors consult The Journal's Rigour and Reproducibility requirements to ensure that their manuscript is suitable for publication: https://physoc.onlinelibrary.wiley.com/pb-assets/hub-assets/physoc/documents/TJP-Rigour-and-Reproducibility-Requirements.pdf

REVISION CHECKLIST:

Check that your Methods section conforms to journal policy: https://jp.msubmit.net/cgi-bin/main.plex?form_type=display_requirements#methods

Check that data presented conforms to the statistics policy: https://jp.msubmit.net/cgi-bin/main.plex?form_type=display_requirements#statistics

Upload a full Response to Referees file. To create your 'Response to Referees' copy all the reports, including any comments from the Senior and Reviewing Editors, into a Microsoft Word, or similar, file and respond to each point, using font or background colour to distinguish comments and responses and upload as the required file type.

Please upload two versions of your manuscript text: one with all relevant changes highlighted and one clean version with no changes tracked. The manuscript file should include all tables and figure legends, but each figure/graph should be uploaded as separate, high-resolution files. The journal is now integrated with Wiley's Image Checking service. For further details, see: https://www.wiley.com/en-us/network/publishing/research-publishing/trending-stories/upholding-image-integrity-wileys-image-screening-service

You may also upload:

- 'Potential Cover Art' for consideration as the issue's cover image
- Appropriate Supporting Information (Video, audio or data set: see https://jp.msubmit.net/cgi-bin/main.plex?form_type=display_requirements#supp).

We look forward to receiving your revised submission.

If you have any queries, please reply to this email and we will be pleased to advise.

Yours sincerely,

Bjorn Knollmann
Senior Editor
The Journal of Physiology

EDITOR COMMENTS

Reviewing Editor:

Thank you for revising your manuscript according to the referee's suggestions. They both found the article largely improved. There are a few minor suggestions for revision and additional information is required to comply with the journal's ethics policy. Thanks.

Senior Editor:

I concur with the reviewing editor's assessment

REFEREE COMMENTS

Referee #1:

The authors have adequately responded to all of my comments.

Referee #2:

Thanks to the authors who have addressed my comments in detail. I have a few minor points for the consideration of the authors below.

1. It would be useful to mention Figure 4 C in the text of the results section.

2. The addition of Appendix Figure A1 is very useful. Purely for aesthetic purposes, the authors could crop out the very small text on top of the image. In most cases the error is very clear from the images but perhaps a line of text in the appendix for confirmation would help the reader e.g. merge errors (cardiomyocyte - cardiomyocyte) shows an example where two different cardiomyocytes have been detected as a single cardiomyocyte.

3. Is the distance map function described in section 2.8 similar to that use previously in cardiac tissue e.g. Caldwell 2014 PMID: 25332206? The values for rabbit and sheep ventricle appear very similar and if this method is the same perhaps this would help validate this automated method?

Referee #3 (ethics review):

Thank you for submitting your manuscript to The Journal of Physiology. The authors have provided sufficient details concerning ethical approvals and sources of animal and human tissue. Relevant citations are provided in the text. Some minor revision is required.

1. Line 196. Please state killed or euthanised in place of sacrificed.

2. Line 197: If available, please provide details on the dose and route of administration of anaesthetic agents in rats (not just concentration).

3. Line 199: State killed or euthanised, not sacrificed.

END OF COMMENTS

Point-by-point response to Reviewing Editor, Senior Editor, and Reviewer 1:

Reviewing Editor:
Thank you for revising your manuscript according to the referee's suggestions. They both found the article largely improved. There are a few minor suggestions for revision and additional information is required to comply with the journal's ethics policy. Thanks.
Senior Editor:
I concur with the reviewing editor's assessment
Referee #1:
The authors have adequately responded to all of my comments.
Answer:
We would like to thank the reviewing editor, senior editor, and Reviewers 1, 2, and 3 for the constructive & fair peer review. We believe the manuscript improved substantially as a result.

Point-by-point response to Reviewer 2:

Reviewer 2:
Thanks to the authors who have addressed my comments in detail. I have a few minor points for the consideration of the authors below.

1. It would be useful to mention Figure 4 C in the text of the results section.
Answer:
Many thanks for pointing that out. We now mention Figure 4C in the results section.

Reviewer 2:
2. The addition of Appendix Figure A1 is very useful. Purely for aesthetic purposes, the authors could crop out the very small text on top of the image. In most cases the error is very clear from the images but perhaps a line of text in the appendix for confirmation would help the reader e.g. merge errors (cardiomyocyte - cardiomyocyte) shows an example where two different cardiomyocytes have been detected as a single cardiomyocyte.
Answer:
Thank you for the suggestion. We have cropped out the text on top of the image and added the requested information to the figure legend.

Reviewer 2:
3. Is the distance map function described in section 2.8 similar to that use previously in cardiac tissue e.g. Caldwell 2014 PMID: 25332206? The values for rabbit and sheep ventricle appear very similar and if this method is the same perhaps this would help validate this automated method?
Answer:
Thank you for pointing this out. Indeed, the values appear surprisingly similar. We now explicitly mention this similarity in the discussion.

Point-by-point response to reviewer 3:

Reviewer 3:
Thank you for submitting your manuscript to The Journal of Physiology. The authors have provided sufficient details concerning ethical approvals and sources of animal and human tissue. Relevant citations are provided in the text. Some minor revision is required.

1. Line 196. Please state killed or euthanised in place of sacrificed.

2. Line 197: If available, please provide details on the dose and route of administration of anaesthetic agents in rats (not just concentration).

3. Line 199: State killed or euthanised, not sacrificed.
Answer:
Thank you for these comments. We have revised the article accordingly.

2nd Revision - Editorial Decision 18-Aug-2025

Dear Mr Greiner,

Re: JP-RP-2025-288557R2 "A Deep Learning-Enabled Toolkit for the 3D Segmentation of Ventricular Cardiomyocytes" by Joachim Greiner, Fabio Frangiamore, Frédéric Sonak, Josef Madl, Thomas Seidel, Peter Kohl, and Eva A. Rog-Zielinska

We are pleased to tell you that your paper has been accepted for publication in The Journal of Physiology.

The last Word (or similar) version of the manuscript provided will be used by the Production Editor to prepare your proof. When this is ready you will receive an email containing a link to Wiley's Online Proofing System. The proof should be thoroughly checked and corrected as promptly as possible.

Authors should note that it is too late at this point to offer corrections prior to proofreading. Major corrections at proof stage, such as changes to figures, will be referred to the Editors for approval before they can be incorporated. Only minor changes, such as to style and consistency, should be made at proof stage. Changes that need to be made after proof stage will usually require a formal correction notice.

All queries at proof stage should be sent to: TJP@wiley.com.

Following copyediting and typesetting the accepted version will be published online.

Yours sincerely,

Bjorn Knollmann
Senior Editor
The Journal of Physiology

IMPORTANT POINTS TO NOTE FOLLOWING YOUR PAPER'S ACCEPTANCE:

If you would like to receive our 'Research Roundup', a monthly newsletter highlighting the cutting-edge research published in The Physiological Society's family of journals (The Journal of Physiology, Experimental Physiology, Physiological Reports, The Journal of Nutritional Physiology and The Journal of Precision Medicine: Health and Disease), please click this link, fill in your name and email address and select 'Research Roundup':
https://www.physoc.org/journals-and-media/membernews

• TRANSPARENT PEER REVIEW POLICY: To improve the transparency of its peer review process, The Journal of Physiology publishes online as supporting information the peer review history of all articles accepted for publication. Readers will have access to decision letters, including Editors' comments and referee reports, for each version of the manuscript as well as any author responses to peer review comments. Referees can decide whether or not they wish to be named on the peer review history document.

• You can help your research get the attention it deserves! Check out Wiley's free Promotion Guide for best-practice recommendations for promoting your work at: www.wileyauthors.com/eeo/guide. You can learn more about Wiley Editing Services which offers professional video, design, and writing services to create shareable video abstracts, infographics, conference posters, lay summaries, and research news stories for your research at: www.wileyauthors.com/eeo/promotion.

• IMPORTANT NOTICE ABOUT OPEN ACCESS: To assist authors whose funding agencies mandate public access to published research findings sooner than 12 months after publication, The Journal of Physiology allows authors to pay an Open Access (OA) fee to have their papers made freely available immediately on publication.

The Corresponding Author will receive an email from Wiley with details on how to register or log-in to Wiley Authors Services where you will be able to place an order.

• You can check if your funder or institution has a Wiley Open Access Account here: https://authorservices.wiley.com/author-resources/Journal-Authors/licensing-and-open-access/open-access/author-compliance-tool.html.

EDITOR COMMENTS

Reviewing Editor:

Thank you for addressing the remaining comments.

Senior Editor:

Thank you for your excellent contribution!
